# Getting used to it? Stress of repeated management procedures in semi-domesticated reindeer

**DOI:** 10.1186/s12917-025-04718-8

**Published:** 2025-04-14

**Authors:** Sebastian G. Vetter-Lang, Nikolaus Huber, Leif Egil Loe, Alina L. Evans, Jouko Kumpula, Per Medbøe Thorsby, Erik Ropstad, L. Monica Trondrud

**Affiliations:** 1 Centre for Food Science and Veterinary Public Health, University of Veterinary Medicine Vienna, Vienna, Austria; 2 Faculty of Environmental Sciences and Natural Resource Management, Norwegian University of Life Sciences, Ås, Norway; 3 Department of Forestry and Wildlife Management, Inland Norway University of Applied Sciences, Campus Evenstad, Elverum, Norway; 4 Natural Resources Institute Finland (Luke), Helsinki, Finland; 5 Hormone Laboratory, Department of Medical Biochemistry and Biochemical Endocrinology and Metabolism Research Group, Oslo University Hospital, Oslo, Norway; 6 Institute of Clinical Medicine, University of Oslo, Oslo, Norway; 7 Faculty of Veterinary Science, University of Life Sciences, Ås, Norway

**Keywords:** Context dependent stress response, Glucocorticoids, Catecholamines, Leukocyte coping capacity, Habituation

## Abstract

**Background:**

Extensive animal production systems, such as reindeer husbandry may represent a system to further study the context dependence of stress responses and the potential implications for animal welfare as research on food animal stress and welfare has so far primarily focused on animals in intensive animal production systems while animals from extensive production systems, such as reindeer, are yet underrepresented. We investigated short- and longer-term stress responses to repeated herding, handling and restraint and its potential effect on animal welfare in semi-domesticated adult female reindeer (*Rangifer tarandus tarandus*). We also assessed seasonal differences and the potential effect of the additional stressor of calf removal using serum concentrations of glucocorticoids (cortisol, cortisone and corticosterone), their precursors (11-desoxcortisol, 17-α-hydroxyprogesterone and deoxycorticosterone) and catecholamine metabolites (metanephrine and normetanephrine) in combination with the immunological stress proxy leukocyte coping capacity (LCC) and rectal temperature. Additionally, we assessed the interconnections among different stress indices and their suitability as stress indicators to evaluate handling-induced stress in reindeer, where rectal temperature, other than serum cortisol levels, emerged as a robust and integrative stress parameter.

**Results:**

Herding, handling, and restraint elicited a marked and seasonally different short-term stress response with higher stress mediator levels in winter. Further, females who had their calf removed shortly after parturition showed increased stress levels based on LCC. The repeated exposure to the same stressors led to a habituation, with decreasing levels of stress indices to the procedure in both seasons. This outcome implies that reindeer females in the present study were able to cope well with repeated manipulations and that this intensification may not compromise animal welfare. Notably, the traditional stress index body temperature correlated with various stress indices encompassing the HPA axis response (cortisol and corticosterone in summer and additionally cortisone and 11-deoxycortisol in winter), the sympathetic-adrenal-medullary system (metanephrine) as well as the immunological response to stress (LCC), in both seasons.

**Conclusion:**

Our results emphasise body (rectal) temperature as a robust and integrative stress parameter in the context of our study. Our findings add to a foundation for evaluating available stress indices in different individual and environmental contexts and may contribute to improved animal management practices aimed at reducing stress levels and enhancing animal welfare.

**Supplementary Information:**

The online version contains supplementary material available at 10.1186/s12917-025-04718-8.

## Background

The improvement of animal welfare is driven by ethical responsibility and economic considerations, especially in modern animal production, and, in many countries, has entered the public policy mainstream [1, 2]. In general, animal management practices can cause significant stress to the animals, affecting their welfare and possibly health, and productivity [3, 4]. The concepts of stress and welfare are multidimensional and complementary. From the animal’s perspective, they are intrinsically related, with increased stress not necessarily indicating decreased welfare, and vice versa, depending on the context [5–7]. The magnitude, duration, and nature of a stress response, along with the animal’s ability to cope based on individual condition and environmental context, are crucial in determining the biological costs or benefits of stress and its potential impact on welfare [8–11]. Arndt and colleagues [12] recently captured the complexity of animal welfare. Consistent with other animal welfare concepts, a central component of their definition is the individual’s capacity to adequately cope with and adapt to internal and external conditions and stimuli [5, 6, 13, 14]. Difficulty or the inability to cope with predictable or unpredictable stressors can cause prolonged elevated stress levels [11, 15]. Therefore, it is essential to quantitatively evaluate the impact of direct human management interventions on individual stress levels within an environmental and seasonal context to better understand their potential effects on animal welfare, productivity, and ultimately animal health [5].

Next to zoo animal research, research on stress and animal welfare has focused primarily on farm animals in intensive systems, providing constant environmental conditions with relatively strict and predictable routines [16]. In contrast, animals in extensive production systems are rather underrepresented and may represent a system to further study the context dependence of stress and linked consequence for animal welfare [17, 18]. Compared to animals in intensive systems, human interactions are infrequent and typically seasonal [19], and may therefore trigger a stronger stress response [17, 20]. Furthermore, many extensively kept animals can face extreme seasonal challenges with harsh environmental conditions and significant fluctuations in food/energy availability, being potential additional stressors. One example of extensive livestock production are reindeer (*Rangifer tarandus*). Reindeer are a socio-economically important livestock, particularly in the subarctic, tundra and boreal regions of North America, Siberia, and Northern Europe, where they are kept as managed but free ranging, semi-domesticated herds [21]. They have a recent domestication history and are genetically similar to wild extant populations [22]. The ongoing change in land use and warming of the Arctic and surrounding areas may necessitate more frequent and intensive reindeer husbandry systems in the future [23]. These changes could involve increased corralling, handling, physical restraint, and transportation, all known as marked stressors [4].

The two central physiological systems to cope with stressors, the sympathetic nervous system (SNS) and the hypothalamic-pituitary-adrenal axis (HPA-axis), are relatively well-studied [24, 25]. Upon the perception of a potential stressor, the SNS is activated within milliseconds, followed by the instant release of noradrenaline from peripheral adrenergic nerves and adrenaline via the sympathetic-adrenal-medullary (SAM) axis, triggering an immediate “fight or flight” response. The HPA axis orchestrates the production and release of glucocorticoids (GCs) to regulate various physiological pathways, adjusting both, up and downregulation of body functions to manage the stress response and support allostasis [26]. Measuring glucocorticoid hormone levels has generally been adopted as a standard procedure to estimate individual stress levels, whereas it has been recently stated that it is required to include several glucocorticoids (i.e., cortisol, cortisone, and corticosterone) to evaluate stress and animal welfare [27]. Additionally, it has been stated that relying solely on GCs to assess stress and welfare is insufficient [28]. Furthermore, several publications highlight that a comprehensive approach, including additional stress/welfare indices such as the activation of the SNS and proxies involving the endocrine-immune interface along with standard physiological measurements such as e.g., body temperature, should be adopted [16, 29–31].

**Fig. 1:**
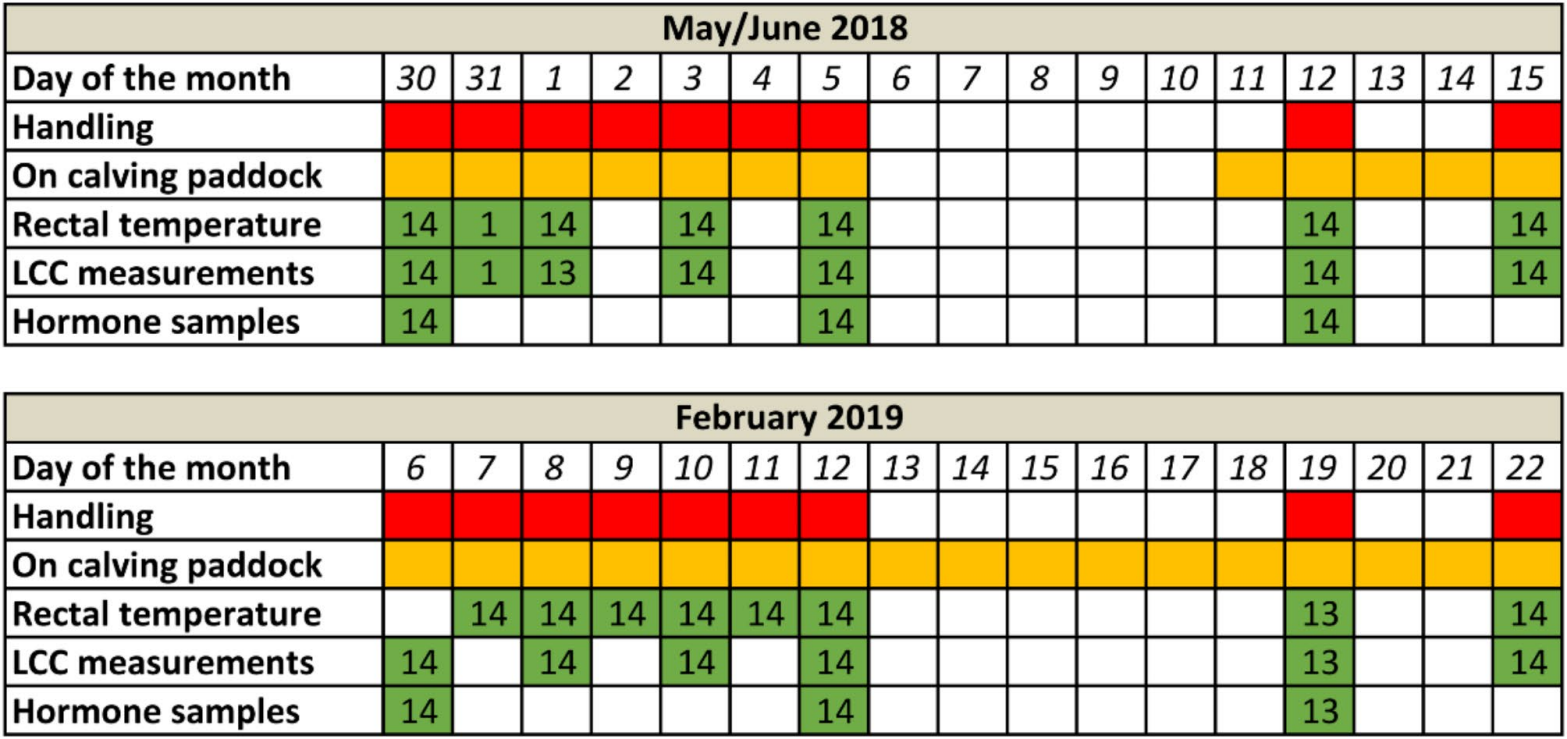
Animal handling and sampling regime of the 14 reindeer females involved in the study

In this study, we repeatedly measured a suite of stress responses in 14 individually marked female reindeer in Northern Finland over a 17-day period in summer and winter (Fig. 1). We aimed to quantitatively assess **i**) the stress level inflicted by herding, handling and restraint, i.e., short-term stress, **ii**) the direction of stress levels when this procedure is repeated over a prolonged period of time, i.e., habituation or sensitization affecting long-term stress, and **iii**) potential seasonal differences in these responses. Moreover, we aimed to **(iv)** assess the potential effect of calf removal (and lactation) on stress levels in semi-domestic reindeer. We further sought to **(v)** gain insights into the interconnections among different stress indices and explore their suitability as stress indicators in the field to evaluate handling induced stress in this species. Hence, to cover the activation of the two main stress axes i.e., the HPA axis and the SNS we included blood serum concentrations of two glucocorticoids (cortisol, corticosterone), the glucocorticoid metabolite cortisone, theprecursors (17-α-hydroxyprogesterone (17-OHP), 11-deoxycortisol (11-DEO), deoxycorticosterone (DOC)) as well as metanephrine (MN) and normetanephrine (NMN), the metabolites of adrenaline and noradrenaline. Cortisol has a different plasma half-life (appr. 60 min.) compared to cortisone and corticosterone (appr. 60–90 min.) [24]. The combined approach of measuring these different glucocorticoids may provide different information during the stress response towards our defined stressor. In combination with the precursor molecules these measures may detect differences in the endogenous regulation of glucocorticoid hormones with repeated stress events or between seasons [24, 32].

In addition, we combined this hormonal approach with an immunological stress proxy, the leukocyte coping capacity [33, 34] being rather a result of the stress response and not directly involved in comparison to the orchestrating hormones and being a more integrative parameter for the individual’s ability to cope with the stress and therefore animal welfare [31]. We further included the classic physiological stress parameter of rectal temperature, which is easy to apply and use in the field and therefor may be of relevance to animal managers in the field [35]. Understanding patterns in stress parameters is crucial as it establishes a better foundation to evaluate available stress indices as well as stressors more effectively.

We predicted that the animal manipulation with capture and handling will trigger a marked stress response including all parameters measured as well as a seasonal difference with increased levels of stress indices during winter, when environmental conditions are more challenging for the animals. Further, we hypothesized that the concentrations of stress mediators and the other stress metrics involved will show an increase in stress level with each sampling event, with an even more pronounced increase during winter. We also predict that the removal of calves is posing a stressor leading to increased levels of stress mediators and a lower capacity to cope with the stress of capture and handling.

Our goal with this study is to contribute to a better understanding of context dependent stress responses leading to the development of sustainable and ethical management practices to reduce stress and thereby, promote animal health and welfare.

## Results

### Effects of capture and handling

**Fig. 2:**
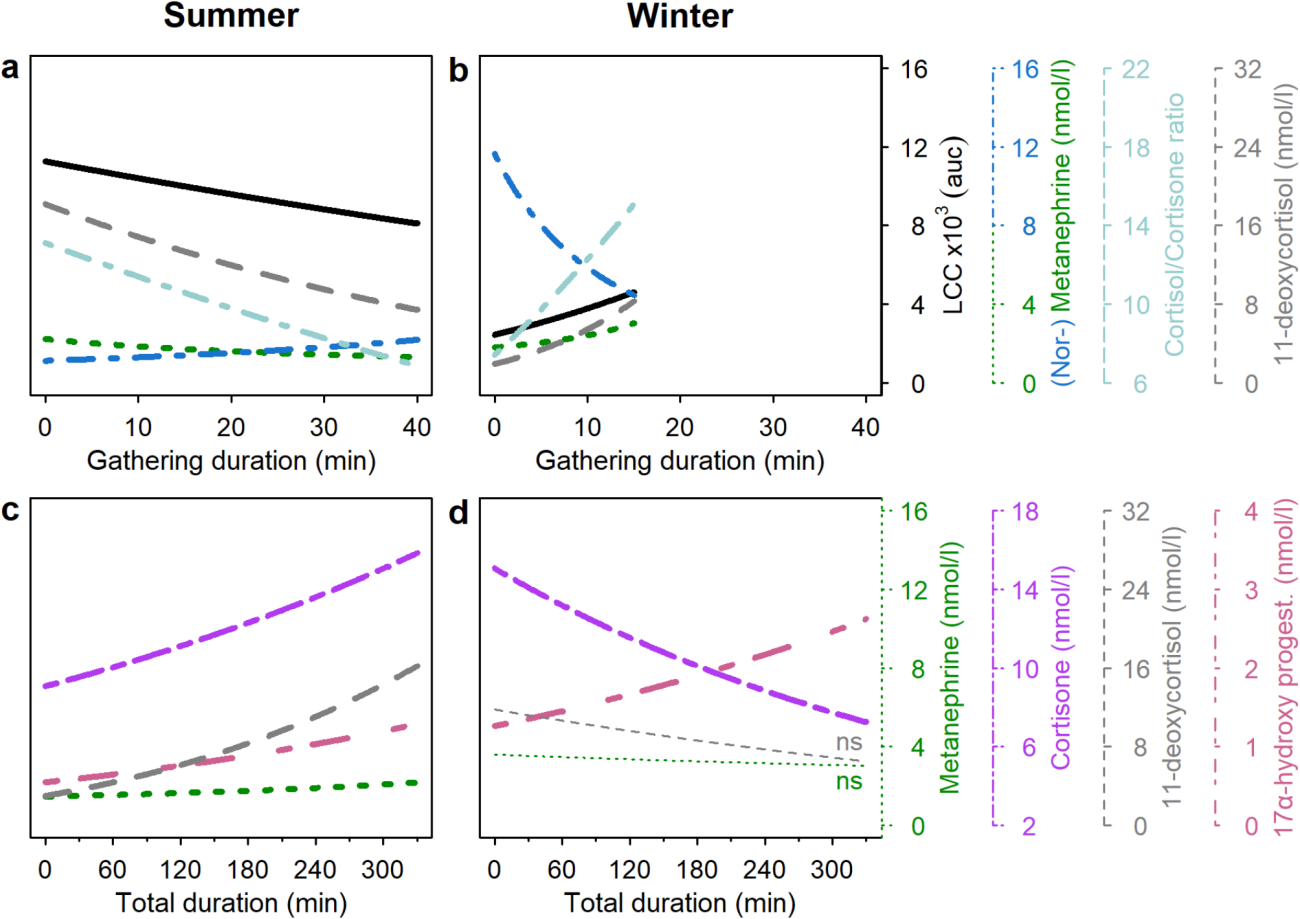
Partial effect plots of the effect of the gathering duration (**a** and **b**) and total duration of animal manipulation (**c** and **d**) on stress indices in summer (**a** and **c**) and winter (**b** and **d**) extracted from the respective best models (see additional Table A1). Regression lines are shorter in (**b**) as the gathering duration in winter did not exceed 15 min whereas it was up to 38 min in summer. Significant effects (i.e., slope significantly different from zero) are indicated by bold lines, non-significant slopes with a thin line annotated with “ns”. *P*-values and other statistical values are given in additional Table A1. Colours and line types of regression lines and y-axes indicate the various stress indices. Only stress indices on which gathering or total duration showed a significant effect in at least one season are shown. The positive effects of gathering duration in winter and of total duration in summer on deoxycorticosterone are not displayed for reasons of clarity. For these effects see additional Table A1

**Fig. 3:**
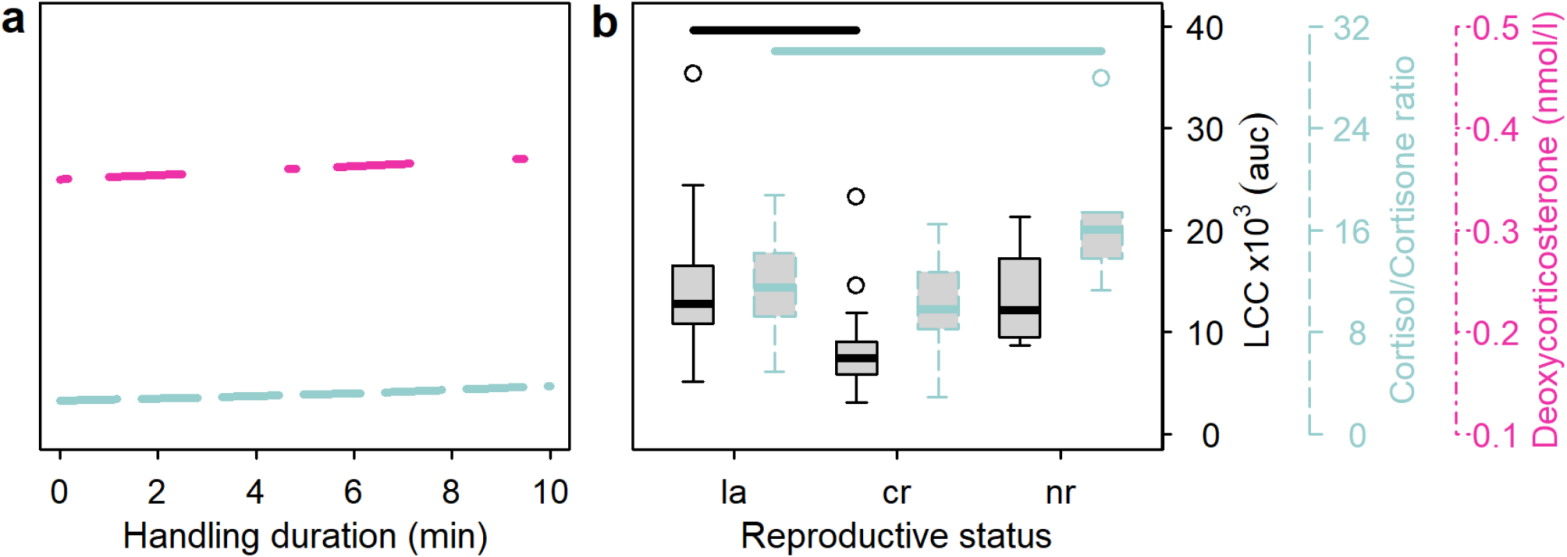
Partial effect plots of the effect of handling duration on deoxycorticosterone and the cortisol/cortisone ratio (**a**), and of reproductive status (la: cow with calf and lactating; cr: calf removed, not lactating, nr: cow did not reproduce, not lactating) on LCC and the cortisol/cortisone ratio (**b**). Only stress indices on which handling duration or reproductive status showed a significant effect are shown. Significant differences between categories in (B) are indicated by horizontal lines. *P*-values and other statistical values on the effects of handling duration and reproductive status are given in the additional tables A1 and A2, respectively. Colours and line types of regression lines and y-axes indicate the various stress indices

Cortisone increased with total duration of animal manipulation in summer but decreased in winter (Fig. 2cd). The cortisol/cortisone ratio decreased with gathering duration in summer but increased with this variable in winter (Fig. 2ab). Further, the cortisol/cortisone ratio increased with handling duration (Fig. 3a). The model on the cortisol/cortisone ratio, however, generally only explained a rather moderate amount of variation as shown by the low ΔAICc of the Null model only containing the intercept (additional Table A1). 11-DEO was affected positively by gathering duration and total duration in summer (Fig. 2ac), whereas gathering duration affected this variable negatively in winter and total duration had no effect in this season (Fig. 2bd). Additionally, handling duration tended to affect 11-DEO positively (additional Table A1). 17-OHP increased with total duration (Fig. 2cd). DOC increased with gathering duration in winter, with the total duration in summer (additional Table A1), and with handling time independent of season (Fig. 3a).

MN was linked to the gathering duration in both seasons but again with contrasting effects (i.e., negative in summer, positive in winter; Fig. 2ab). Additionally, MN was positively associated with the total duration in summer (Fig. 2cd). Contrasting the effects on MN, NMN was positively affected by gathering duration in summer (Fig. 2a) but negatively in winter (Fig. 2b). Further, there were trends for positive effects of handling duration (additional Table A1) and lack of reproduction on NMN (i.e., that non-reproducing females showed higher NMN levels compared to lactating ones; additional Table A2).

LCC was significantly affected by duration of gathering in both seasons, however, with different effects in summer and winter (negative in summer, positive in winter; Fig. 2ab). Rectal temperature tended to increase with gathering duration and decrease with the total duration of animal manipulation.

### Effects of repeated handling on stress indices

**Fig. 4:**
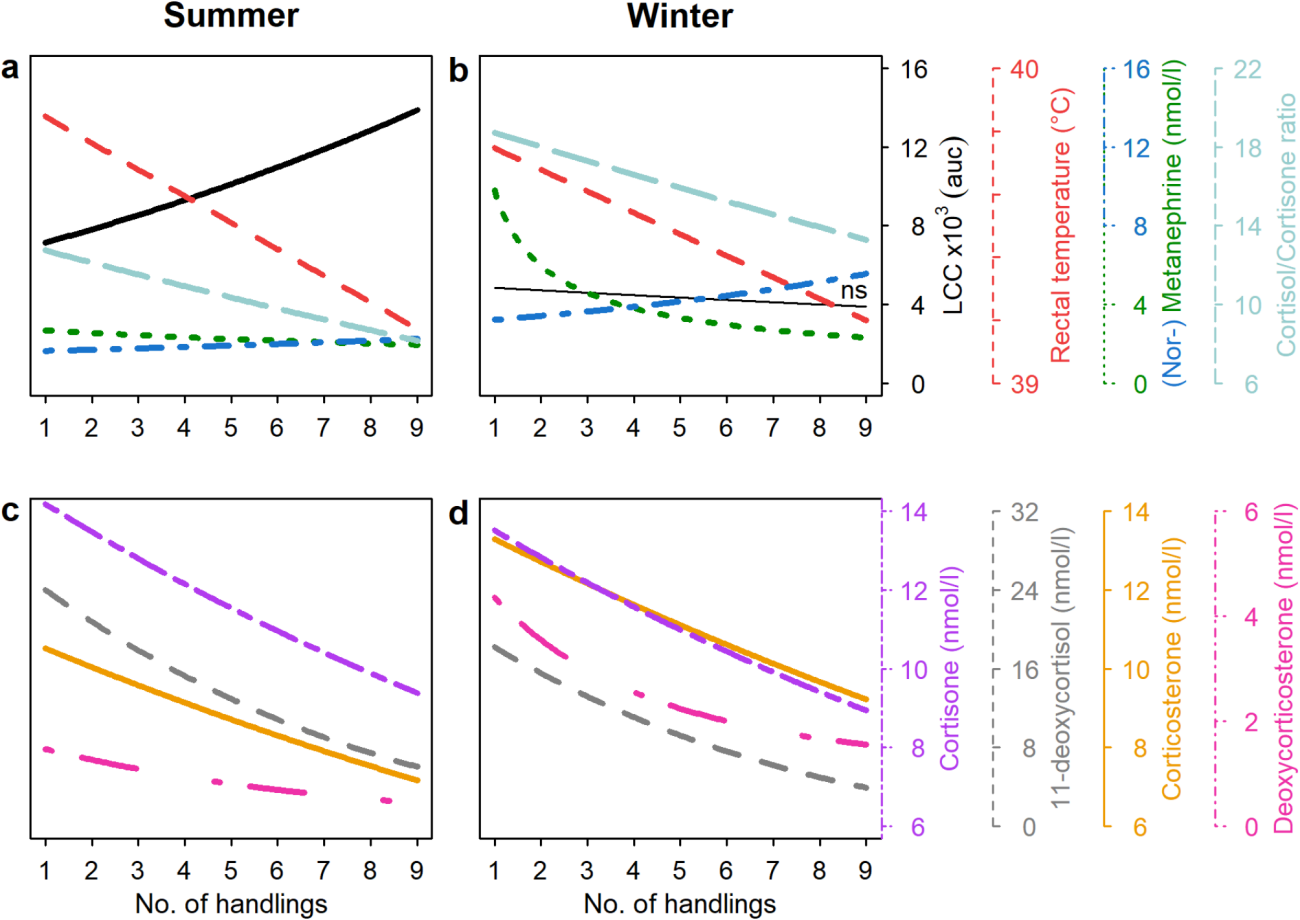
Partial effect plots of the effect regarding the number of previous handlings on stress indices representing HPA axis- and SNS-activity as well as LCC in summer (**a** and **c**) and winter (**b** and **d**) extracted from the respective best models (see additional Table A1). Significant effects (i.e., slope significantly different from zero) are indicated by bold lines, the non-significant effect of the number of handlings on LCC with a thin line annotated with “ns”. *P*-values and other statistical parameters are given in additional Table A1. Colours and line types of regression lines and y-axes indicate the various stress indices. Only stress indices where the number of previous handlings showed a significant effect in at least one season are displayed

Cortisone decreased with repeated handling events in both seasons as did the cortisol/cortisone ratio, 11-DEO, DOC, and corticosterone (Fig. 4ab). However, as for cortisol and the cortisol/cortisone ratio, also for corticosterone the Null model was within a ΔAICc < 10 (additional Table A1). Further, cortisol and 17-OHP showed trends for the same negative effect of repeated handling.

Also, MN decreased with repeated handlings in both seasons while NMN increased with repeated handlings (Fig. 4ab). Repeated handlings affected LCC positively only in summer but showed no effect in winter (Fig. 4ab). The model on rectal temperature revealed a season independent decrease with repeated handlings (Fig. 4ab).

### Effects of reproductive status in summer on stress indices

Non-reproducing females showed a higher cortisol/cortisone ratio compared with lactating females with calf (Fig. 2b). A trend for such an effect could further be observed for cortisol and 17-OHP (additional Table A2). However, as for cortisol/cortisone ratio above also for cortisol the Null model was within a ΔAICc < 10 (additional Table A1). Females from which the calf was removed exhibited a lower LCC response compared with lactating females with calf in summer, while non-reproducing females did not show any difference from lactating individuals regarding LCC levels. (Fig. 2b). Summary outputs of the summer models analysing the effect of reproductive status are given in additional Table A2.

### Seasonal effects on stress indices

Based on models containing season as the sole explanatory variable none of the main hormones representing HPA-axis activity, i.e., cortisol, cortisone or corticosterone showed differences in mean levels, except for a trend towards significance in the cortisol/cortisone ratio (Table 1). All other parameters differed between seasons regarding their mean levels (Table 1). However, many models from the complete analysis including the additional explanatory variables, selection analyses revealed interactive effects between season and various explanatory variables (see Table 1 and below; for details see additional Table A1 showing model selection tables (showing all models within a ΔAICc > 2 plus the respective null-model only containing the intercept), ANOVA results and summary outputs from models without intercepts (see methods) for all cross-seasonal analyses).

**Table 1:** Effect of season on the measured stress indices collected from 14 female reindeer during repeated handling procedures

Parameter		Summer		Winter		Effect of season	
	*n*	(mean ± sd)	(range)	(mean ± sd)	(range)	on value^1^	via int. effects^2^
Cortisol (nmol/l)	83	115.5 ± 53.3	16.0–242.0	135.3 ± 62.3	16.0–246.0	0.123	No
Cortisone (nmol/l)	83	12.0 ± 4.5	5.3–26.0	11.5 ± 4.6	4.4–23.0	0.675	Yes
Cortisol/Cortisone ratio	83	10.1 ± 5.0	1.8–26.4	12.5 ± 6.4	2.1–27.7	0.069	Yes
Corticosterone (nmol/l)	83	10.8 ± 8.7	1.5–40.0	13.3 ± 8.1	0.9–31.0	0.165	No
11-deoxycortisol (nmol/l)	83	15.4 ± 15.9	0.6–77.0	6.6 ± 5.6	0.1–21.0	0.001	Yes
17α-hydroxy progest. (nmol/l)	83	1.3 ± 1.8	0.2–10.0	2.1 ± 1.4	0.2–5.7	0.021	No
Deoxycorticosterone (nmol/l)	83	1.4 ± 1.7	0.1–7.5	0.5 ± 0.5	0.1–2.1	0.003	Yes
Metanephrine (nmol/l)	83	1.8 ± 0.7	1.0–4.3	2.7 ± 1.0	1.4–6.1	< 0.001	Yes
Normetanephrine (nmol/l)	83	2.3 ± 1.4	0.6–7.8	4.2 ± 1.7	2.1–10.0	< 0.001	Yes
Rectal temperature (°C)	196	39.9 ± 0.6	38.5–41.7	39.4 ± 0.5	37.8–41.0	< 0.001	No
LCC (auc)	167	11,043 ± 5653	2644–28,076	3727 ± 1759	1018–11,162	< 0.001	Yes

^1^ Based on models containing season as the sole explanatory variable

^2^ Indicating significant effects of season via interactive effects with other independent variables (for details see additional Table A1)

### Links between stress indices

The pairwise comparisons between the measured metabolites and stress indices showed correlations of varying strength among GCs within the stress response. We could identify strong positive correlations between cortisol, corticosterone, and the cortisol and corticosterone precursors 11-DEO, 17-OHP, and DOC in both seasons (correlation coefficients (r) ranging from 0.51 to 0.89; additional figure A1, A2). Cortisone showed slightly weaker positive correlations with the other GCs and their precursors in winter (r from 0.40 to 0.62; additional figure A2) and no correlations at all with these variables in summer (all *r* < 0.19; except for cortisol, additional figure A1). A positive link between MN and NMN could only be identified in winter as between MN and cortisol (additional figure A2). Correlations of MN with 17-OHP and DOC, respectively, could only be identified in summer (additional figure A1). Apart from the link with MN in winter, NMN was not correlated to any of the other variables (additional figure A1, A2).

A negative correlation between LCC and rectal temperature was identified in summer (*r* = -0.31; additional figure A1), whereas the link between these two variables was reversed in winter (*r* = 0.34; additional figure A2). Further, for LCC trends were identified for a negative link with cortisone in summer (*r* = -0.34; additional figure A1) and a positive link with DOC in winter (*r* = 0.35; additional figure A2). Apart from LCC, rectal temperature was positively correlated with MN (*r* = 0.53 and 0.55 in winter and summer, respectively), cortisone (*r* = 0.43 and 0.30 in winter (only trend) and summer, respectively), and corticosterone in both seasons (*r* = 0.54 and 0.41 in winter and summer, respectively; additional figures A1, A2) and additionally with cortisol (*r* = 0.56) and its precursor 11-DEO (*r* = 0.50) only in winter (additional figure A2).

For further details on test-statistics, marginal R²-values (extracted utilising the R-package performance [36]) correlation coefficients, and *p*-values see additional figures A1 (summer) and A2 (winter).

## Discussion

In this study, we show that reindeer elicited stress responses to each handling event, but, contrary to our prediction, the magnitude of the response was attenuated with repeated handling events within a season, which, to our knowledge, has not been assessed in semi-domesticated reindeer. In line with our prediction, we show a seasonal difference in stress responses in reindeer with comparatively higher stress mediator levels in winter than in summer. Based on the immunological measure of stress (leukocyte coping capacity, LCC), females of which had their calf removed showed a decreased capacity to cope with the stress events, which could not have been detected by only using the hormonal approach. Further, we found an effect of body weight on cortisol levels and seasonally differing links between the single stress indices.

Each sampling event of the study reflects the circumstances of a typical reindeer handling procedure where processes such as checking of ownership, marking of calves and anthelmintic treatment is only feasible after animals have been gathered, corralled, and subsequently restrained for handling. It is critical to note that the absolute levels and correlations among the measured stress parameters are obtained from animals exposed to a stressful situation with variations in stressor duration. Thus, these values do not represent baseline or peak levels. The durations of gathering and handling as well as the overall duration of animal manipulation (i.e., from gathering to blood sampling) were included in all full statistical models to account for potential duration effects. Our study only includes female reindeer and it is likely that the male stress responses may differ from females at different reproductive stages [37].

### General levels of stress indices in response to animal manipulation

In the scientific literature the reliability of several steroid immunoassays has been questioned because of the lack of specificity for single GCs and of matrix effects [38, 39]. The application of LC-MS to analyse GC hormones and precursors in this study provided the necessary specificity to avoid overestimating the concentrations and reliably discriminate the abundance of certain GCs (e.g., cortisol vs. corticosterone). The main GC present in our study species was cortisol but also corticosterone was present, although in much lower concentrations. Mean cortisol levels were similar or higher compared to other studies in captured/handled or adrenocorticotropin challenged “semi-domesticated” reindeer *(R. t. tarandus)* [40–42] and Svalbard reindeer (*R. t. platyrhynchus)* [30, 43], confirming that the animals in our study are exhibiting a stress-related activation of the HPA axis in response to the management procedure, as predicted. Only Trondrud et al. [30], offer insights into the additional glucocorticoids and their precursors in Svalbard reindeer, showing similarities with our results from winter. Stress-induced cortisol levels in our study were positively associated with body mass, whereas the scientific literature provides positive, negative or no relationship between glucocorticoid levels and body weight [44–46]. A recent metanalysis highlights a direct relation of GC levels with metabolic rate [47]. Given this context, this outcome in our study could be interpreted as to heavier individuals, presumably being in a better body condition, especially during winter, can afford higher stress induced cortisol levels, highlighting the importance of the individual context in connection with stress. This may enable them to better manage the associated (e.g., energetic) trade-offs and support allostasis more robustly [8, 9]. However, given the low delta AIC of the null model versus the best model for this outcome, which also applies to cortisol/cortisone ratios and corticosterone levels this result should be taken with caution. Future studies may also include the measurement of glucocorticoid-binding globulins (CBGs) to assess both free and bound glucocorticoids. The “free hormone hypothesis” posits that only unbound, free hormones are biologically active, while CBGs prevent tissue exposure. Therefore, incorporating CBG measurements could enhance the interpretation of GC concentrations and stress levels across different seasons or individual variations, such as body mass [48, 49].

Mean catecholamine metabolite levels also showed a marked, acute stress response in our study individuals but were about half of the levels reported for Svalbard reindeer [30]. This may be explained by the different capture method of animals in the latter study, which involved high physical activity (i.e., animals have been chased with snow mobiles), and differences in handling procedure and thus sampling time after stressor onset [35]. Moreover, (sub-) species specific differences in the orchestration of the stress response, physiological strategies to cope with short-term stress, and grade of domestication cannot be ruled out [50–52]. Mean LCC levels for female reindeer in this study were comparable with levels in roe deer (*Capreolus capreolus*), which were captured and handled without sedation or anaesthesia [34]. However, they were notably lower compared with other wildlife species such as brown bears (*Ursus arctos* [53]) or rhinoceros (*Ceratotherium simum simum* [54]). This is, to the best of our knowledge, the first study to report LCC levels for reindeer, and our results add to previous studies showing species-specific differences in LCC levels [31]. Interestingly, LCC levels revealed differences in the context of reproductive status. Reindeer females whose calves were removed showed significantly lower LCC levels compared to individuals with calves or non-lactating females who had not reproduced. Irrespective of the time point (early vs. late weaning) the artificial removal of calves is stressful for a female and her offspring [55] and can increase anxiety and depressive like symptoms [56, 57]. LCC has also been shown to be a reliable proxy for psychological stress in vertebrates [58–60]. The decreased LCC levels in females whose calves have been removed indicate higher stress levels and, overall, can be seen as a diminished capacity to cope with the additional stress of handling [31]. However, given the small sample size per reproductive status those results as well as the lack of evidence from most other stress indices should not be overinterpreted. Our study’s mean rectal temperatures align with those reported in captured/handled wild Svalbard reindeer [30, 43], as well as semi-domestic reindeer [42].

### Effects of (repeated) animal manipulation on stress levels in a seasonal context

In contrast to our predictions, we found no seasonal difference in mean levels for the three primary hormones representing HPA-axis activity (i.e., cortisol, cortisone, corticosterone), corroborating other publications reporting no seasonal differences in serum or plasma cortisol levels in reindeer [40, 61]. However, a study by Nilssen and colleagues [62] reported higher plasma cortisol levels in summer compared to fall and winter, aligning with our observation of doubled levels of the cortisol precursor 11-DEO during summer. The inclusion of measuring of faecal glucocorticoid metabolites may provide additional information, especially as this can be performed non-invasively and has been validated for reindeer in the context of handling procedures [63]. Interestingly, we also found significant seasonal interactive effects with stressor durations and inverted responses to isolated components of animal manipulation, i.e., durations of gathering, handling, and the total duration of the management procedure. The most striking difference was observed in cortisone levels where the increase in total duration of animal manipulation led to significantly increasing levels during summer and the reverse in winter. Cortisone is biologically inactive, and levels are regulated via the activity of the enzymes 11β-hydroxysteroid dehydrogenase (11β-HSD) type 1 and 2 [27, 32]. 11β-HSD 1 catalyses the conversion of cortisone into cortisol whereas type 2 transformers cortisol into cortisone. The cortisol/cortisone ratio therefore allows to estimate 11β-HSD activity. The biological relevance of 11β-HSD 2 lies in its role in preventing cortisol from binding to mineralocorticoid receptors. The observed seasonal differences in and seasonally different effects on cortisol precursors, cortisone, and the cortisol/cortisone ratio may therefore signify a seasonal shift in the endogenous corticosteroid regulation in response to stress, underlining the importance of including the environmental and/or seasonal context when assessing stress responses. This might further suggest a varying seasonal prioritization of mineralo- and glucocorticoid functions and a seasonal modification of the physiological process to cope with stressors [24, 25, 32].

We observed higher MN and NMN levels during winter as well as seasonally different effects of gathering duration and total duration on both catecholamine metabolites and MN, respectively. Several animal studies corroborate the utility of catecholamine metabolites, particularly MN, as indicators of stress-induced activation of the SNS and SAM system [64, 65], including research on reindeer [30]. In contrast to our results, Larsen and colleagues [66] did not observe significant seasonal variations in adrenaline or noradrenaline levels among female Norwegian reindeer. However, their study animals were accustomed to blood sampling and handling and were likely not showing an acute short-term stress responses comparable to animals in our study. Furthermore, despite a lack of overall significance two out of the four animals investigated by Larsen and colleagues showed an increase in noradrenaline levels in February [66], coinciding with winter samples in our study. One plausible explanation for the increased catecholamine metabolite levels in winter may be an enhanced sensitivity to stressors with a more pronounced activation of the stress systems during this time of the year [67]. Alternatively, catecholamine, and noradrenaline levels in particular, could be increased in winter due to cold exposure and change in metabolic demands as shown for humans [68, 69] and goats [70]. However, these explanations are not mutually exclusive as it is highly likely that multiple parallel endogenous factors contribute to the observed seasonal differences in catecholamine metabolites. Similar to GC-levels, also MN and NMN levels were influenced in opposing directions by the different components of animal manipulation. MN levels decreased with gathering duration in summer but vice versa in winter and NMN levels increased with gathering duration in summer but decreased in winter. This dynamic suggests that a longer gathering duration may benefit the animals with a decrease in SAM activation during summer (being not pushed as hard and avoid heat stress) whereas a shorter gathering duration would be beneficial in this regard during winter. This again highlights different sensitivities to stress between seasons, which is further supported by the increase of MN with the total duration of animal manipulation during summer but no such effect during winter. Surprisingly, despite being closest to the actual blood sampling, the duration of direct handling only resulted in an increase NMN but not MN.

We also found significantly decreased LCC levels during winter indicating either higher stress levels or a reduced capacity to cope with the stress of being manipulated during this time of the year [33, 71]. Similarly, European roe deer exhibited decreased LCC levels during winter and it was suggested to avoid capture and handling during winter to reduce stress levels and improve welfare outcomes [31]. However, it is not possible to disentangle the direct effect of stress on LCC from potential seasonal changes in immune function [72, 73] and such assumptions should therefore be taken with caution. Studies conducted on fishes have offered evidence indicating seasonal fluctuations in the respiratory burst activity of neutrophil/heterophil granulocytes [74, 75], although in fish this effect might have been caused by variations in body temperature. In our study, mean rectal temperatures were not different between seasons, even though in approximately 30% of winter- and 85% of summer handling events, the study animals were hyperthermic approaching heat stress (i.e., showed rectal temperatures more than 2 °C higher than the normal core body temperature of ~ 39 °C [76]), which can be linked to the acute stress response of animal manipulation as well as different grades of physical activity [77]. Nevertheless, the rectal temperatures observed during handling in summer indicate that, despite being well adapted to extreme ambient temperatures to avoid either hypo- or hyperthermia, reindeer may be at risk of heat stress during handling which should be especially considered during summer and seasonally unusual ambient temperatures [43, 78]. Although, we could only identify trends towards significance for potential effects of gathering and total duration on rectal temperature here, these trends fall in line with this assumption as gathering tended to affect rectal temperature positively whereas they seemed to “cool off” with increasing total duration (i.e., caused by longer waiting times in the coral between gathering and handling).

Except for 17-OHP, all hormones and their precursors linked with the HPA-axis decreased either significantly (cortisone, corticosterone, 11-DEO and DOC levels) or with a trend towards significance (cortisol) with the number of previous handlings during both seasons not aligning with our prediction. Also, MN levels representing SAM activity followed this dynamic. This can be interpreted as a general decrease of stress levels with each capture/handling procedure and that animals are habituating to the repeated management procedure [79]. This is further supported by the decrease in rectal (body) temperature as well as an increase in LCC which shows a decrease in stress levels during summer [65, 80–82]. Notably, the observed habituation effect in summer did not manifest permanently and the eight-month interval without handling between summer and winter session was sufficiently long to negate the previous habituation. Nevertheless, upon regular exposure to the procedure, animals exhibited habituation once more towards the stressor. Here we would also like to note that the animals in the herd are weighed monthly (excluding summer months) and are therefore, to a certain degree, used to human presence and being manipulated. Hence, the observed stress levels and habituation to the repeated handling procedures may not be representative for reindeer being manipulated only once or twice per year. Further, the animals were fed ad libitum with pellets (in addition to natural forage) during the period of data collection, which may have contributed as a “positive experience” and thereby fostered the habituation to the repeated manipulation [82]. Opposing to the observed decrease of the other stress indices, NMN levels increased with the number of previous handling events. There is evidence from the literature that variations in noradrenaline levels associated with stressor application are incongruent with decreasing corticosteroid levels in the context of habituation to a stressor [83]. This might be because noradrenaline is a key mediator of various cognitive functions promoting the encoding of stress-context related (emotional) memory information [84–86]. Furthermore, it was hypothesised that increased noradrenaline levels after repeated exposure to a stressor may leave the organism better prepared to cope with impending stressors [87]. Thus, our finding of increasing NMN levels along with decreases in all other stress parameters in response to repeated animal manipulation may reflect adaption rather than an increase in stress level and likely indicates habituation and a shift in physiological strategy to cope with the stressor.

### Links between stress indices

The three hormones representing the stress-related activation of the HPA axis, namely cortisol (including its precursors), cortisone, and corticosterone, were strongly and positively correlated in both seasons. In contrast, the associations between MN and NMN varied seasonally, exhibiting correlation only during winter. This might be due to their different functions, i.e., that the SNS remains active even during periods of rest, ensuring cardiovascular performance, whereas adrenaline (SAM) responses are more intricately tied to stress and the reactions of the HPA axis rather than those of the SNS [88, 89]. This is supported by the links of MN with cortisol, corticosterone and the two cortisol precursors which could not be shown for NMN.

Notably, rectal temperature exhibited correlations with various stress indices encompassing the HPA axis response (cortisol and corticosterone in summer and additionally cortisone and 11-DEO in winter), the SAM system (MN) as well as the immunological response to stress (LCC), in both seasons. This emphasizes rectal temperature as a robust and integrative stress parameter. Stress induced changes in body temperature (hyperthermia) are reported to be tuned to stressor intensity and increases can occur already within seconds. Further, it has been stated that body temperature may be indicative of both, short- (seconds of stressor onset) and mid-term stressors (stressor duration up to several days) [35]. Interestingly, LCC levels were correlated with rectal temperature but showed opposite patterns in summer and winter: in summer, LCC decreased (indicating higher stress levels) with increasing rectal temperature whereas it increased in winter with increasing rectal temperature. We speculate that the decreased summer LCC levels may indicate the combined impact of heat stress along with handling and restraint stress. However, we have no plausible explanation for the relationship between LCC and body temperature during winter, other than the possibility that capture related heat stress might be less critical in winter, as the low air temperatures may allow animals to dissipate excess heat more rapidly. Except for a negative trend with decreasing cortisone levels during summer, LCC was not significantly correlated with any of the hormonal parameters. This finding confirms previous work in cervids, i.e., in roe deer in free-ranging [34] and captive conditions [31] as well as other mammalian- [53, 54] and avian- wildlife species [90, 91]. Our results are therefore in line with other studies suggesting LCC may represent different physiological aspects, with different kinetics and response latencies and large individual variation. However, LCC may reflect a more integrative response to a multitude of stress signals in the organism, which potentially explains the absence of direct correlation with classical stress parameters in our and other studies [31]. Additionally, our sampling regime represents one point-in-time within the stress response and the relation between stress indices is likely to be different in dependence of the sampling time during the stress response [35].

The observed significant associations among individual stress metrics, particularly the stronger and more numerous links between stress mediators observed during winter, may suggest a tighter integration and coordination of the central stress axes, namely the HPA-, SAM- axis and the SNS, during winter months. This synchronization could serve to mitigate the allostatic load induced by the stress response and prevent allostatic overload in a seasonal context [29, 92, 93].

## Conclusions

Unsurprisingly, our study shows that the stressors of herding, handling and restraint cause a marked short-term stress response in female reindeer. The duration of components of animal manipulation had varying effects on stress proxies. These effects, however, should not be overinterpreted, as it is not possible to clearly disentangle their impact on stress levels assessed from a single sampling time point at the end of the procedure, given the consecutive nature of the stressors. To better evaluate these individual components (gathering vs. handling and restraint), future studies should consider collecting samples prior to and after each component, ideally with an automated sampling device. This approach may provide clearer insights considering the differing release and reaction dynamics of stress indices, especially of hormones, and allow for a better characterisation and interpretation of the dynamic of stress levels during the entire period of animal manipulation. Additionally, the measurement of faecal glucocorticoid metabolites may provide additional information to interpret serum hormone concentration [63]. The frequently used plasma cortisol measurements did not provide significant insights, which may in part also be attributed to our study design with a single sampling time point. However, in practice and in the field, this is the most probable scenario when reindeer herders can collect a sample to assess stress levels in their animals in order to gather evidence to further improve their animal handling practices reducing stress levels.

Interestingly, the levels of hormonal stress mediators decreased with the repeated exposure to the same animal manipulation, indicating a habituation to the procedure, which was confirmed by the additional non-hormonal proxies for stress. We therefore conclude that the animals in this study were able to cope well with repeated manipulations and further that this intensification may not compromise animal welfare in this context. However, a longer break (several months) between intensive herding and handling sessions potentially negates previous habituation. Our study also reveals that exposure to the same stressor leads to seasonally differing hormonal and stress-related immunological responses, with a more pronounced stress related activation of the HPA and SAM axes as well as the SNS in winter. This implies a potentially higher sensitivity to stress and its associated biological costs in winter. Further, the additional application of LCC measures revealed higher stress levels in females where the calf was removed, which would go undetected when only using the classical hormonal approach. Overall, the combined application and assessment of multiple hormonal stress indicators in combination with non-hormonal stress indices, i.e. LCC and rectal temperature, allowed a robust assessment of stress levels to repeated manipulation. Moreover, the results underscore the importance of considering individual and environmental/seasonal contexts when evaluating stress levels, as the linked biological costs and impacts on animal welfare may vary significantly.

We hope our findings will be considered in the future and contribute to better assessment of animal management practices to reduce stress levels and further improve animal welfare.

## Methods

### Study animals and area

All procedures on reindeer were carried out in accordance with the ARRIVE guidelines and authorized by the Animal Experiment Board of the Regional State Administrative Agency, Finland (license ESAVI/3857/04.10.07/2017). The study was performed at the Kutuharju Reindeer Research Facility (Kaamanen, Northern Finland, 69° 8’ N, 26° 59’ E, https://www.luke.fi/en/research/research-infrastructures/kutuharju-reindeer-research-platform) in June 2018 and February 2019. The study system consists of a herd of ~ 100 animals, belonging to the Reindeer Herders’ Association. The herding management includes keeping reindeer in two summer pastures (~ 13.8 and ~ 15 km^2^) between calving and rutting, and in a winter enclosure (~ 15 km^2^) and a calving paddock to give birth in spring. The area consists of open birch (*Betula* spp.*)* and pine (*Pinus sylvestris*) forest with small lakes and wetlands [94]. The animals in the herd are weighed monthly between September and April each year (excluding summer months), with some variability in timing and capture rates between years.

### Handling procedures

During the period 31 May – 15 June 2018, and between 6 and 23 February 2019, 14 females were subjected to daily handling and sample collection over 7 days, followed by a 6-day break, and another two days of handling (see Fig. 1). The same animals were sampled in summer and winter. During the handling period the study animals were confined to the calving paddock (~ 0.3 km^2^) but were released to the larger summer pasture in between handling periods. In winter, the reindeer were kept in the calving paddock during the break between handling periods. Each morning, the reindeer were herded (by all-terrain vehicle in summer and by snow mobile in winter) into a corral (~ 60 m^2^), then individually, or in mother-calf pairs (summer only), guided into a smaller corral (~ 10 m^2^) before walking into an indoor handling facility. Each reindeer first walked onto a floor scale to be weighed (± 0.5 kg), before being transferred onto a wall-mounted restraining device for blood sampling. While restrained, animals were subjected to blood sampling from the left jugular vein (females and calves) and rectal temperature measurements. Hereby, between 5 and 9 ml of blood were collected using a BD vacutainer system using serum tubes and heparin and rectal temperature was taken by inserting a handheld veterinary thermometer 5–10 cm into the rectum while the animal was bled. We used single-use thermometer sleeves coated with a lubricant to avoid cross-contaminations. After handling, the reindeer were released back into the calving paddock. During the daily handling periods, reindeer were fed ad libitum with pellets in addition to natural forage in the paddock. In terms of reproductive management, calves were separated from their mothers 2–3 weeks after birth in May, which deviates from the standard practice for the herd, which is that calves will stay with their mothers until they are slaughtered in autumn or separate gradually from them after weaning. Removal of calves in spring only occurs in exceptional cases when a calf is hurt or is unlikely to survive until autumn slaughter.

### Hormone assays

Blood samples were kept unfrozen (i.e., at ambient temperature between 5 and 15 °C, for both seasons) until all animals were handled each day. Separated plasma (centrifuged at 3000 rpm for 15 min) was then kept frozen at -20 °C until further analyses. Metanephrines (MN and NMN), glucocorticoids and precursor steroids (cortisol, cortisone, corticosterone, 11-deoxycortisol (11-DEO), deoxycorticosterone (DOC), and 17α-hydroxy progesterone (17-OHP)) were analysed by liquid chromatography tandem mass spectrometry (LC-MS/MS) at the Hormone Laboratory, Oslo University Hospital, Norway accredited according to NS-EN ISO/IEC 17025:2017 for hormonal measurements in humans together with samples collected in a separate study. Quantification limits were 0.2 nmol/L (NMN), 0.1 nmol/L (MN), 0.5 nmol/L (cortisol), 0.2 nmol/L (corticosterone), 0.2 nmol/L (11-DEO), 0.71 nmol/L (DOC) and 0.2 nmol/L (17-OHP). The analytical CV% ranged from 6 to 15% with an accuracy ranging between 90 and 110% for all steroid hormones. Individual samples collected on the same day were analysed together [30]. Due to funding constraints hormone assays were performed on only three of the blood samples per individual and season (i.e., the ones of the first handling day, an intermediate handling day, and towards the end of the handling period in each season respectively; see Fig. 1).

### Leukocyte coping capacity (LCC)

LCC measurements from heparinized full blood samples were analysed immediately after collection. As these analyses were labour and time intense (appr. 60 min per sample) they were carried out every second handling day (Fig. 1) as previously published [34, 53]. In brief, unstimulated blood chemiluminescence levels, indicating baseline levels of reactive oxygen species (ROS), were measured by transferring 10 µl of heparinized whole blood into a silicon antireflective tube (Lumivial, Berthold Technologies, Bad Wildbad, Germany) containing 90 µl of 10^− 4^ mol l^− 1^ luminol (VWR International, Sweden) and 10 µl of PBS. For full blood chemiluminescence measurement in response to a secondary challenge, a parallel tube was prepared with the same procedure but containing 10 µl of 10^− 5^ mol l^− 1^ phorbol 12-myristate 13-acetate (PMA; VWR International, Stockholm, Sweden) instead of PBS. Blood chemiluminescence for each tube was assessed every 5 min for a total of 30 s over a period of 50 min and expressed in relative light units (RLU), using a portable high sensitivity chemiluminometer (Junior LB 9509, Berthold Technologies, Bad Wildbad, Germany). All measurements were carried out inside a closed room at the handling facility ensuring stable conditions above 15 °C. When not in the chemiluminometer, tubes were protected from light and incubated at 37 °C using a metal bead bath (Minitüb, Tiefenbach, Germany).

### Statistical analysis

All models described below contained animal id as random effect to account for repeated measurements of the same individuals and were checked for deviations from normality by means of histograms and qq-plots. Where necessary data were boxcox-transformed [95]. This was the case for all variables except for rectal temperature where this was not necessary. All models on transformed data showed no deviations from normality. Variance inflation was checked for all models (excluding interactions) but did not reach relevant levels in any of the models. All analyses were performed in R 4.2.2 [96].

To assess the effect of herding and handling, separate full models were constructed for LCC, rectal temperature, MN, NMN, cortisol, cortisone, cortisol-cortisone ratio, corticosterone, 11-DEO, 17-OHP, and DOC, containing the respective variable as dependent variable and the following independent variables, respectively: total duration (start of animal gathering, i.e., when the herding was initiated, until the exact time of blood sampling, i.e., when blood sampling was completed), gathering duration (start of animal gathering until all animals were inside the waiting pen), handling duration (time between when the animal entered the handling facility and blood sampling), number of previous handlings in the respective season, body mass, as well as the pairwise interaction of all those variables with season. From these models, model selection based on the AICc (Akaikes information criterion corrected for small sample size [97, 98] was conducted using the function “dredge” in the R-package MuMIn [99]. From the respective best model, i.e., the one showing the lowest AICc, parameter estimates and their respective 95%-confidence intervals (CIs) were extracted. To facilitate direct comparison across all levels of main effects and interactions we fitted models without intercept, from which parameter estimates and their respective 95%-CIs were extracted. To analyse the effect of reproductive status in summer (i.e., lactating female with calf, female from which calf was removed, or non- reproducing female) an additional mixed effects model was created for each dependent variable with summer data only including only explanatory variables that showed an effect in summer (based on the 95%-CIs from models without common intercept). To show the effect of a given variable on the different stress indices in a comparable manner the different effects of a variable were displayed in summarised partial effects plots in the results that show effects on the original, i.e., back-transformed scales, but do not contain data points or measures of uncertainty to facilitate readability of the plots. To provide this information we created separate partial effects plots for each effect that show the respective effect on the linear, i.e., transformed scale, including its standard error and the data points (additional figures A3, A4, A5, A6, A7).

Given the many identified interactive effects between season and other explanatory variables, we tested for differences in seasonal means of each stress parameter using linear mixed effects models containing season as the sole explanatory variable.

Finally, pairwise links between all response variables have been investigated in single mixed effects models (R-package nlme [100]). *P*-values from these pairwise models were corrected for multiple testing using the Benjamini-Hochberg method [101]. The analysis of these pairwise links between dependent variables were performed for summer and winter data separately.

## Electronic supplementary material

Below is the link to the electronic supplementary material.

**Supplementary Material 1**: **Additional Figure A1**: Correlations between stress indices in summer. The upper diagonal shows scatterplots of the raw data, in case of a significant correlation (*p* ≤ 0.05), a solid regression line, and, in case of a trend for a correlation (0.1 > *p* > 0.05), a dashed regression line. The lower diagonal shows the following statistical values: the marginal r² (extracted utilising the R-package performance), the correlation coefficient r (i.e., the square root of the marginal r² multiplied by either − 1 or + 1, depending on the direction of the effect), the *p*-value of the correlation and the sample size n.

**Supplementary Material 2**: **Additional Figure A2**: Correlations between stress indices in winter. The upper diagonal shows scatterplots of the raw data, in case of a significant correlation (*p* ≤ 0.05), a solid regression line, and, in case of a trend for a correlation (0.1 > *p* > 0.05), a dashed regression line. The lower diagonal shows the following statistical values: the marginal r² (extracted utilising the R-package performance), the correlation coefficient r (i.e., the square root of the marginal r² multiplied by either − 1 or + 1, depending on the direction of the effect), the *p*-value of the correlation and the sample size n.

**Supplementary Material 3**: **Additional figure A3**: Partial effect plots of the effect of the gathering duration on the different stress indices including its respective standard error as well as the data points, all on the linear scale of boxcox-transformed data. Back-transformed predictions on the original scale can be seen in Fig. 2ab.

**Supplementary Material 4**: **Additional figure A4**: Partial effect plots of the effect of total duration on the different stress indices including its respective standard error as well as the data points, all on the linear scale of boxcox-transformed data. Back-transformed predictions on the original scale can be seen in Fig. 2cd.

**Supplementary Material 5**: **Additional figure A5**: Partial effect plots of the effect of the handling duration on the different stress indices including its respective standard error as well as the data points, all on the linear scale of boxcox-transformed data. Back-transformed predictions on the original scale can be seen in Fig. 2a.

**Supplementary Material 6**: **Additional figure A6**: Partial effect plots of the effect of the reproductive status on the different stress indices (means ± standard errors) as well as the data points, all on the linear scale of boxcox-transformed data. Boxplots of back-transformed data on the original scale can be seen in Fig. 2b.

**Supplementary Material 7**: **Additional figure A7**: Partial effect plots of the effect of the number of handlings on the different stress indices including its respective standard error as well as the data points, all on the linear scale of boxcox-transformed data. Back-transformed predictions on the original scale can be seen in Fig. 4.

**Supplementary Material 8**: **Additional Table A1**: For all stress indices analysed three tables are shown: **(i)** the model selection table including all models within a ΔAICc ≤ 2 plus the null-model only containing the intercept (left column), **(ii)** estimates and their 95%-confidence intervals (CI) from the best model as well as its marginal (without random effects) and conditional (including random effects) R²-values as an indicator for the amount of variance explained (middle column; variables were the 95%-CI did not include zero were highlighted in green and bold; main effects were not highlighted when the 95%-CI of the interaction with season showed did not include zero), and **(iii)** the estimates and their 95%-confidence intervals from the best model but without common intercept (right column). The latter was done to identify seasonal slopes of those variables, which showed a significant interaction with season. Seasonal slopes for which the 95%-CI did not include zero were highlighted in green and bold; main effects already highlighted in the model with common intercept (middle column) were not highlighted again. In model selection tables (left column) names of independent variables were number coded for reasons of clarity. The legend with the number codes can be found at the very bottom.

**Supplementary Material 9**: **Additional table A2**: For all stress indices the summary outputs of the summer models are shown, which have been generated to analyse the effects of reproductive status in summer and were based on effects identified for summer in the cross-seasonal models (additional Table A1). Results showing the potential effects of reproductive status were printed black whereas results from independent variables, which were included to correct for their effect in summer identified in the cross-seasonal model, were printed in grey. Significant *p*-values of seasonal independent variables were highlighted in green and bold; trends of seasonal independent variables were highlighted in green and italics.

## Data Availability

All data underlying this publication have been made publically available (10.34876/phqe-sf42).
