# Supplementary material for: Getting used to it? Stress of repeated management procedures in semi-domesticated reindeer
Source: BMC Vet Res. 2025 Apr 14;21:268. doi: 10.1186/s12917-025-04718-8 (PMC11995495; doi:10.1186/s12917-025-04718-8)
Supplement: Supplementary file 2 — Supplementary Material 2: Additional Figure A2: Correlations between stress indices in winter. The upper diagonal shows scatterplots of the raw data, in case of a significant correlation (p ≤ 0.05), a solid regression line, and, in case of a trend for a correlation (0.1 > p > 0.05), a dashed regression line. The lower diagonal shows the following statistical values: the marginal r² (extracted utilising the R-package performance), the correlation coefficient r (i.e., the square root of the marginal r² multiplied by either − 1 or + 1, depending on the direction of the effect), the p-value of the correlation and the sample size n. [file 12917_2025_4718_MOESM2_ESM.pdf]

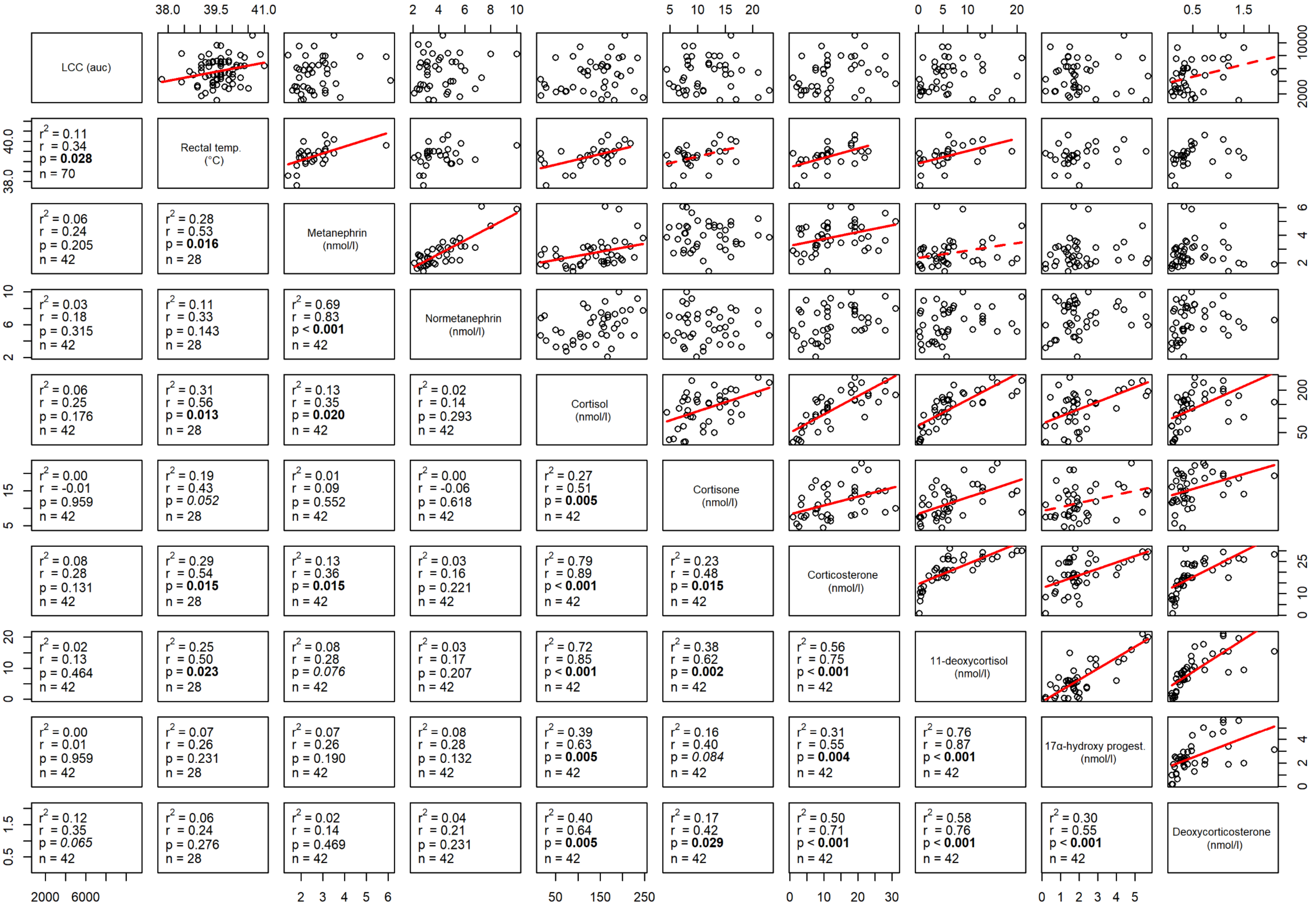

Supplementary Figure S2: Correlations between stress indices in winter. The upper diagonal shows scatterplots of the raw data, in case of a significant correlation ($p \leq 0.05$), a solid regression line, and, in case of a trend for a correlation ($0.1 > p > 0.05$), a dashed regression line. The lower diagonal shows the following statistical values: the marginal r^2 (extracted utilising the R-package performance), the correlation coefficient r (i.e., the square root of the marginal r^2 multiplied by either -1 or +1, depending on the direction of the effect), the p-value of the correlation and the sample size n.
