# Supplementary figures and images for: Getting used to it? Stress of repeated management procedures in semi-domesticated reindeer
Source: BMC Vet Res. 2025 Apr 14;21:268. doi: 10.1186/s12917-025-04718-8 (PMC11995495; doi:10.1186/s12917-025-04718-8)

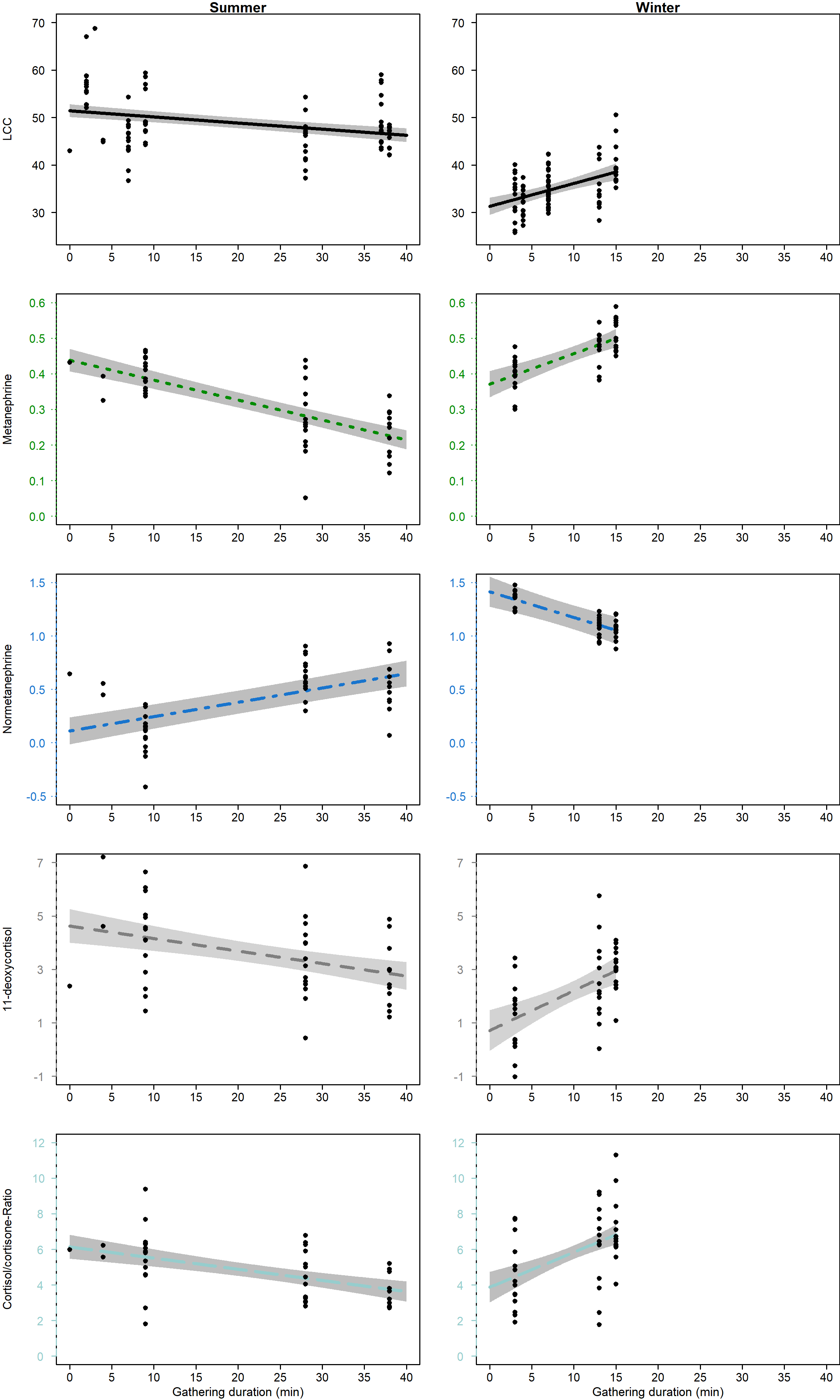

Supplement: Supplementary file 3 — Supplementary Material 3: Additional figure A3: Partial effect plots of the effect of the gathering duration on the different stress indices including its respective standard error as well as the data points, all on the linear scale of boxcox-transformed data. Back-transformed predictions on the original scale can be seen in Fig. 2ab. [file 12917_2025_4718_MOESM3_ESM.tif]

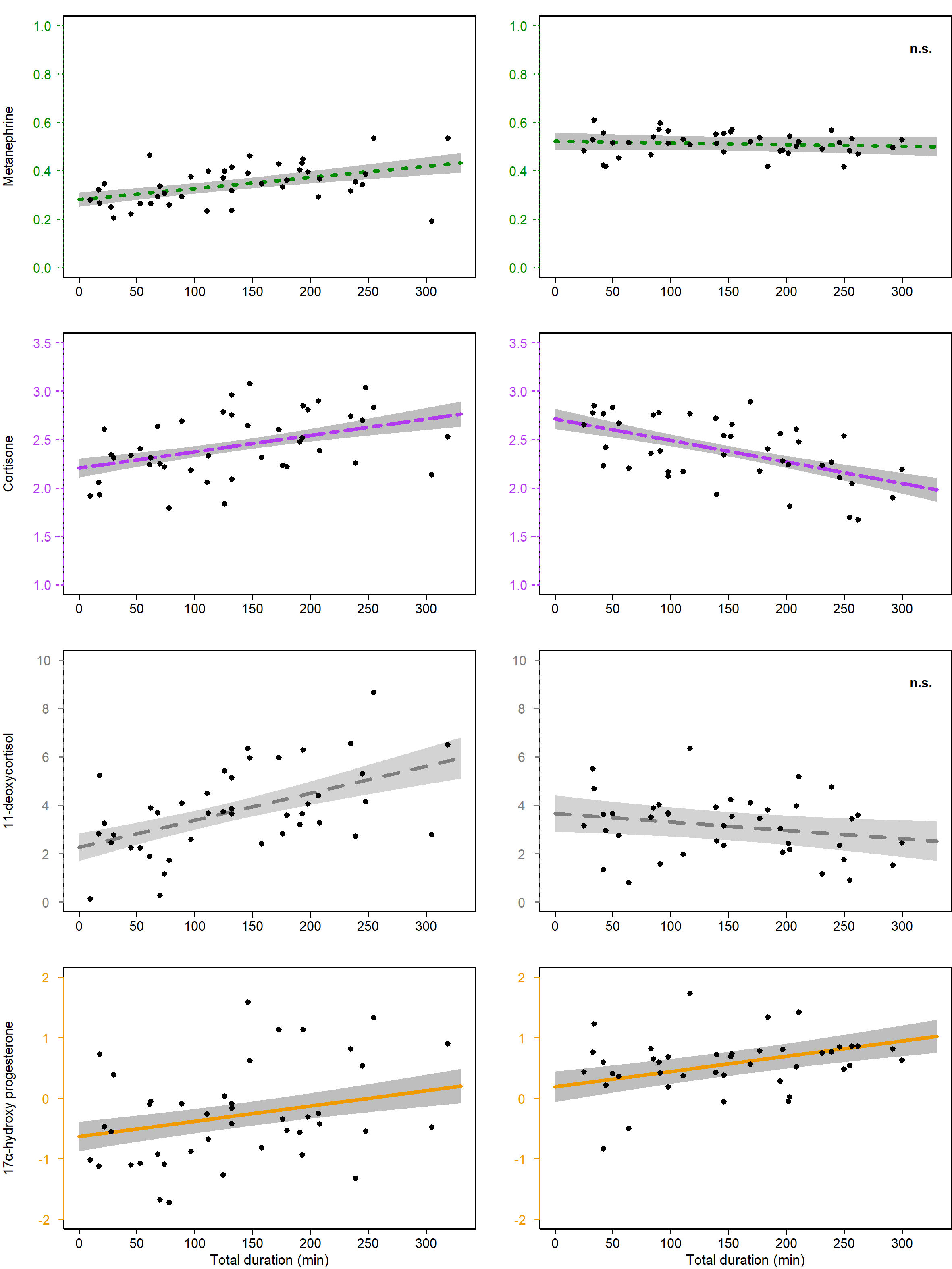

Supplement: Supplementary file 4 — Supplementary Material 4: Additional figure A4: Partial effect plots of the effect of total duration on the different stress indices including its respective standard error as well as the data points, all on the linear scale of boxcox-transformed data. Back-transformed predictions on the original scale can be seen in Fig. 2cd. [file 12917_2025_4718_MOESM4_ESM.tif]

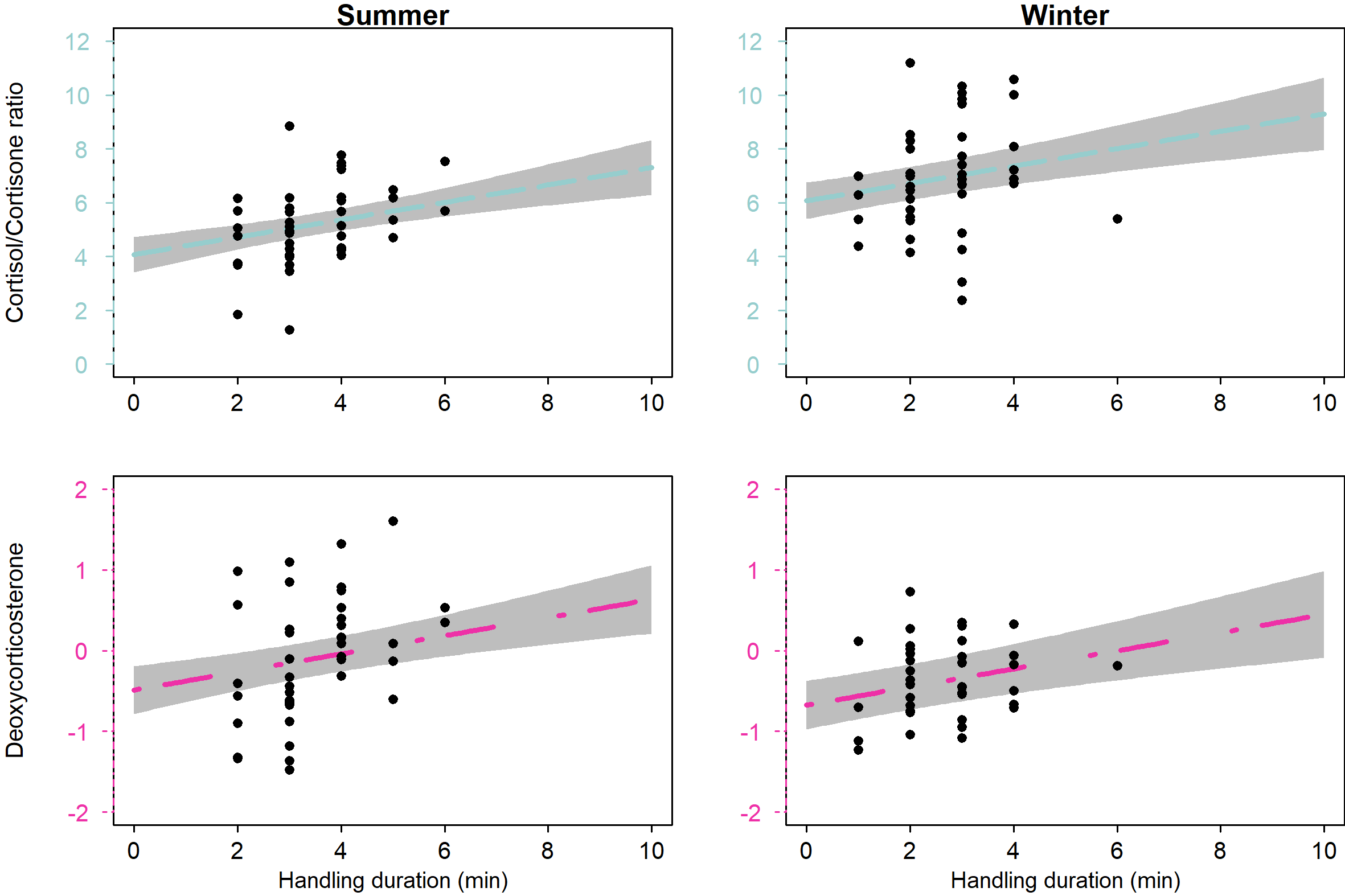

Supplement: Supplementary file 5 — Supplementary Material 5: Additional figure A5: Partial effect plots of the effect of the handling duration on the different stress indices including its respective standard error as well as the data points, all on the linear scale of boxcox-transformed data. Back-transformed predictions on the original scale can be seen in Fig. 2a. [file 12917_2025_4718_MOESM5_ESM.tif]

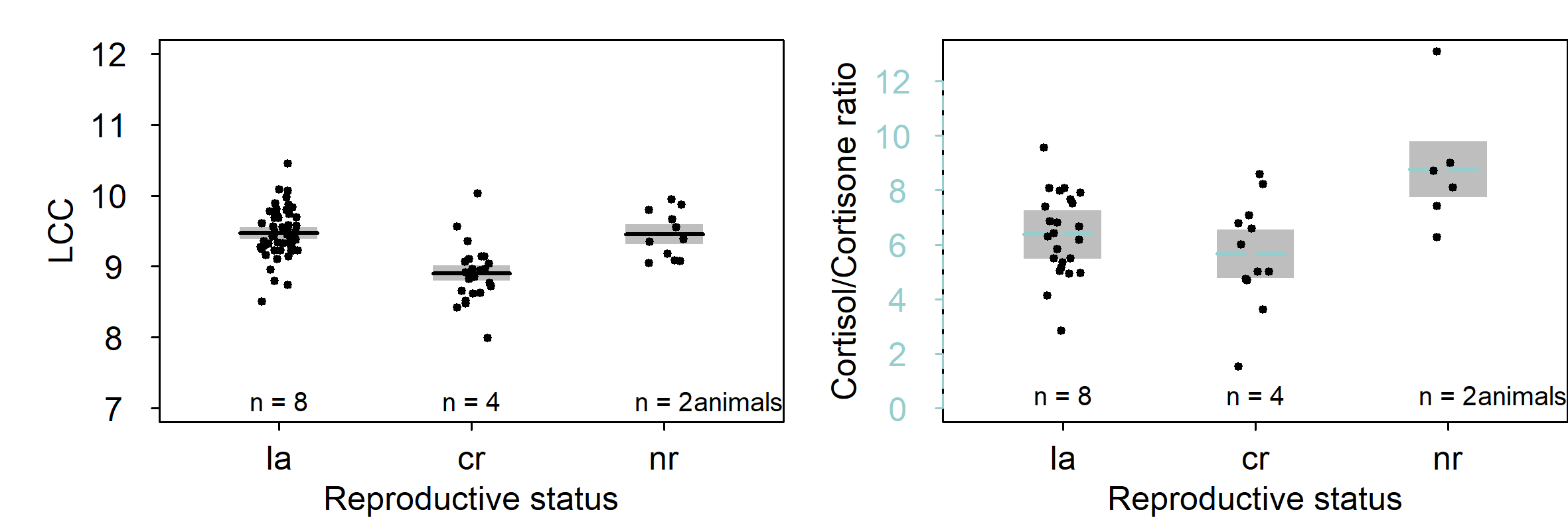

Supplement: Supplementary file 6 — Supplementary Material 6: Additional figure A6: Partial effect plots of the effect of the reproductive status on the different stress indices (means ± standard errors) as well as the data points, all on the linear scale of boxcox-transformed data. Boxplots of back-transformed data on the original scale can be seen in Fig. 2b. [file 12917_2025_4718_MOESM6_ESM.tif]

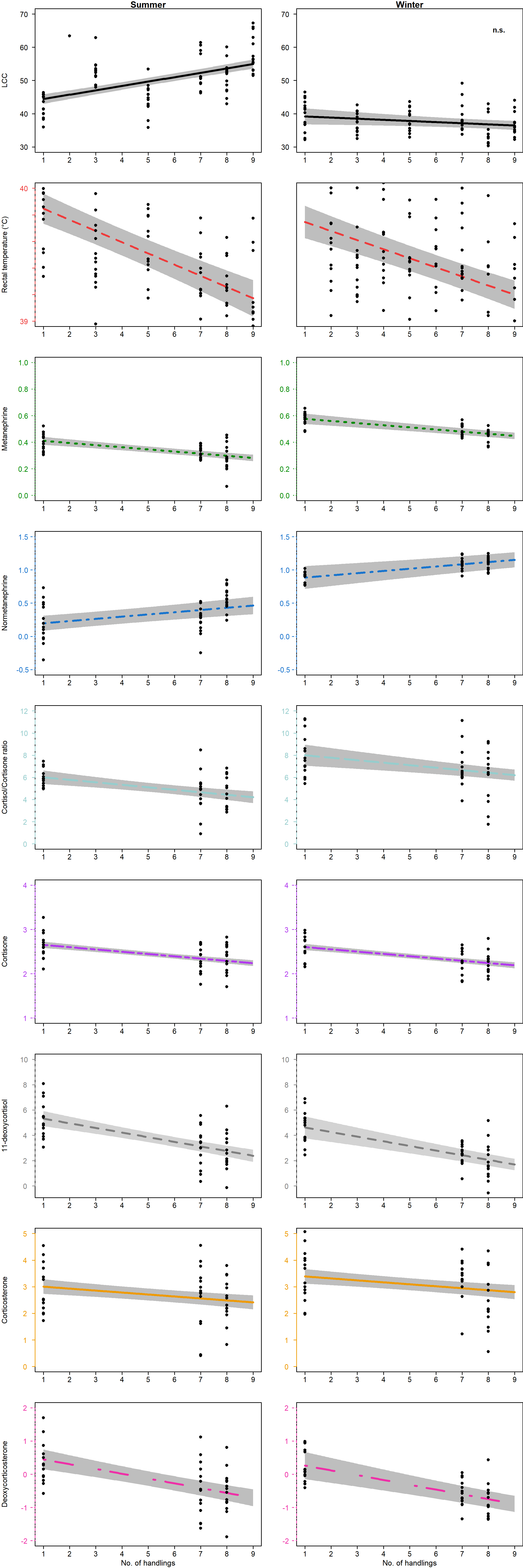

Supplement: Supplementary file 7 — Supplementary Material 7: Additional figure A7: Partial effect plots of the effect of the number of handlings on the different stress indices including its respective standard error as well as the data points, all on the linear scale of boxcox-transformed data. Back-transformed predictions on the original scale can be seen in Fig. 4. [file 12917_2025_4718_MOESM7_ESM.tif]
