# Supplementary material for: Getting used to it? Stress of repeated management procedures in semi-domesticated reindeer
Source: BMC Vet Res. 2025 Apr 14;21:268. doi: 10.1186/s12917-025-04718-8 (PMC11995495; doi:10.1186/s12917-025-04718-8)
Supplement: Supplementary file 8 — Supplementary Material 8: Additional Table A1: For all stress indices analysed three tables are shown: (i) the model selection table including all models within a ΔAICc ≤ 2 plus the null-model only containing the intercept (left column), (ii) estimates and their 95%-confidence intervals (CI) from the best model as well as its marginal (without random effects) and conditional (including random effects) R²-values as an indicator for the amount of variance explained (middle column; variables were the 95%-CI did not include zero were highlighted in green and bold; main effects were not highlighted when the 95%-CI of the interaction with season showed did not include zero), and (iii) the estimates and their 95%-confidence intervals from the best model but without common intercept (right column). The latter was done to identify seasonal slopes of those variables, which showed a significant interaction with season. Seasonal slopes for which the 95%-CI did not include zero were highlighted in green and bold; main effects already highlighted in the model with common intercept (middle column) were not highlighted again. In model selection tables (left column) names of independent variables were number coded for reasons of clarity. The legend with the number codes can be found at the very bottom. [file 12917_2025_4718_MOESM8_ESM.pdf]

Model selection tables

LCC_both seasons (n = 167)

	df	logLik	AICc	ΔAICc	weight
2+4+5+8+10	8	-527.16	1071.23	0.00	0.19
1+2+4+5+8+10	9	-526.25	1071.64	0.41	0.15
2+4+5+6+8+10	9	-526.85	1072.86	1.63	0.08
(Null)	3	-620.57	1247.29	176.06	0.00

Rectal temperature_both seasons (n = 196)

	df	logLik	AICc	ΔAICc	weight
1+2+4+5+6+7+8+11	11	-90.50	204.43	0.00	0.05
1+2+3+4+5+6+7+8+11	12	-89.83	205.36	0.93	0.03
1+2+4+5+6+7+8	10	-92.20	205.58	1.16	0.03
1+2+4+5+6+7+11	10	-92.37	205.93	1.50	0.02
1+2+4+5+6+7	9	-93.60	206.17	1.75	0.02
1+2+4+5+7+8	9	-93.61	206.19	1.76	0.02
1+2+3+4+5+6+7+11	11	-91.39	206.21	1.78	0.02
1+2+4+5+7	8	-94.80	206.37	1.94	0.02
(Null)	3	-123.19	252.50	48.07	0.00

Metanephrine_both seasons (n = 83)

	df	logLik	AICc	ΔAICc	weight
2+4+5+6+8+11	9	94.41	-168.35	0.00	0.17
1+2+4+5+6+8+11	10	95.33	-167.60	0.75	0.12
2+4+5+6+8+10+11	10	94.73	-166.41	1.94	0.06
(Null)	3	54.76	-103.21	65.14	0.00

Normetanephrine_both seasons (n = 83)

	df	logLik	AICc	ΔAICc	weight
1+2+3+4+5+7+8	10	-6.23	35.52	0.00	0.10
1+2+3+4+5+7+10	10	-6.53	36.12	0.59	0.07
1+2+4+5+7+8	9	-8.10	36.66	1.13	0.05
1+2+3+4+5+7+8+9	11	-5.63	36.97	1.45	0.05
2+4+5+8	7	-10.85	37.19	1.67	0.04
(Null)	3	-40.91	88.12	52.60	0.00

Cortisol_both seasons (n = 83)

	df	logLik	AICc	ΔAICc	weight
1+4	5	-446.97	904.72	0.00	0.07
1+3+4	6	-446.12	905.35	0.63	0.05
1	4	-448.49	905.50	0.78	0.05
(Null)	3	-451.99	910.28	**5.57**	0.00

Cortisone_both seasons (n = 83)

	df	logLik	AICc	ΔAICc	weight
4+5+6+11	7	-18.86	53.22	0.00	0.14
2+4+5+6+11	8	-18.26	54.47	1.26	0.08
2+4+5+6+8+11	9	-17.04	54.54	1.32	0.07
2+4+5+6+10+11	9	-17.22	54.91	1.69	0.06
1+4+5+6+11	8	-18.58	55.10	1.88	0.06
(Null)	3	-36.58	79.46	26.25	0.00

Cortisol/Cortisone Ratio (n = 83)

	df	logLik	AICc	ΔAICc	weight
2+3+4+5+8	8	-171.90	361.74	0.00	0.07
2+3+4+5+6+8	9	-171.24	362.94	1.20	0.04
1+2+3+4+5+8	9	-171.41	363.29	1.55	0.03
2+3+4+5+8+10	9	-171.55	363.57	1.83	0.03
1+3	5	-176.42	363.63	1.88	0.03
(Null)	3	-180.25	366.79	**5.05**	0.01

Corticosterone_both seasons (n = 83)

	df	logLik	AICc	ΔAICc	weight
4+5	5	-126.77	264.33	0.00	0.06
2+4	5	-127.05	264.89	0.56	0.04
4	4	-128.21	264.94	0.61	0.04
1+4	5	-127.20	265.18	0.85	0.04
3+4+5	6	-126.17	265.44	1.11	0.03
1	4	-128.66	265.83	1.50	0.03
5	4	-128.79	266.09	1.76	0.02
2+4+5	6	-126.50	266.11	1.79	0.02
(Null)	3	-130.18	266.67	**2.34**	0.02

11-deoxycortisol_both seasons (n = 83)

	df	logLik	AICc	ΔAICc	weight
2+3+4+5+6+8+11	10	-157.52	338.10	0.00	0.10
2+4+5+6+8+11	9	-159.08	338.63	0.52	0.08
4+5+6+11	7	-161.93	339.36	1.26	0.05
(Null)	3	-175.22	356.75	18.65	0.00

17α-hydroxy progesterone_both seasons (n = 83)

	df	logLik	AICc	ΔAICc	weight
4+5+6	6	-98.36	209.82	0.00	0.05
2+4+5+6+10+11	9	-95.16	210.78	0.96	0.03
4+5+6+11	7	-97.69	210.87	1.05	0.03
5+6	5	-100.08	210.95	1.13	0.03
2+4+5+6	7	-97.76	211.02	1.20	0.03
1+4+5+6	7	-97.79	211.08	1.26	0.03
3+4+5+6	7	-97.97	211.44	1.62	0.02
2+4+5+6+8+11	9	-95.53	211.53	1.71	0.02
2+4+5+6+11	8	-96.91	211.76	1.94	0.02
(Null)	3	-113.98	234.27	24.45	0.00

Deoxycorticosterone (n = 83)

	df	logLik	AICc	ΔAICc	weight
1+2+3+4+5+6+8+11	11	-97.04	219.80	1.26	0.05
3+4+5+6+10+11	9	-99.68	219.83	1.29	0.05
4+5+6+11	7	-102.43	220.36	1.81	0.04
3+4+5+6+11	8	-101.21	220.37	1.83	0.04
2+4+5+6+8+11	9	-100.01	220.48	1.94	0.04
(Null)	3	-113.99	234.29	15.75	0.00

Legend

1 bodymass
2 gathering duration
3 handling duration
4 no. of handlings
5 season
6 total duration
7 season : bodymass
8 season : gathering duration
9 season : handling duration
10 season : no. of handlings
11 season : total duration

Estimates and confidence intervals (CI) from best model

LCC_both seasons (n = 167)

	estimate	lower 95%-CI	upper 95%-CI
Intercept	46.168	43.122	49.215
season_winter	-13.519	-16.979	-10.060
no.handlings	1.313	0.887	1.740
dur.gathering	-0.129	-0.212	-0.046
season_winter : no. of handlings	**-1.654**	**-2.373**	**-0.934**
season_winter : gathering duration	**0.612**	**0.232**	**0.992**

$R^2_m = 0.60$; $R^2_c = 0.72$

Rectal temperature_both seasons (n = 196)

	estimate	lower 95%-CI	upper 95%-CI
Intercept	40.619	39.242	41.996
season_winter	-0.074	-0.362	0.213
no. of handlings	**-0.084**	**-0.121**	**-0.048**
gathering duration	0.005	-0.001	0.011
handling duration	-0.002	-0.054	0.051
total duration	-0.001	-0.001	0.000
bodymass	-0.009	-0.026	0.008
season_winter : no. of handlings	0.016	-0.030	0.062
season_winter : handling duration	-0.008	-0.062	0.046

$R^2_m = 0.18$; $R^2_c = 0.47$

Metanephrine_both seasons (n = 83)

	estimate	lower 95%-CI	upper 95%-CI
Intercept	0.437	0.363	0.511
season_winter	0.008	-0.089	0.105
no. of handlings	**-0.016**	**-0.024**	**-0.008**
gathering duration	-0.006	-0.008	-0.004
total duration	0.000	0.000	0.001
season_winter : gathering duration	**0.014**	**0.007**	**0.021**
season_winter : total duration	**-0.001**	**-0.001**	**0.000**

$R^2_m = 0.46$; $R^2_c = 0.72$

Normetanephrine_both seasons (n = 83)

	estimate	lower 95%-CI	upper 95%-CI
Intercept	0.633	-0.878	2.144
season_winter	3.003	1.559	4.448
no. of handlings	**0.033**	**0.005**	**0.061**
gathering duration	0.013	0.007	0.019
handling duration	0.036	-0.002	0.074
bodymass	-0.010	-0.029	0.010
season_winter : gathering duration	**-0.037**	**-0.059**	**-0.015**
season_winter : bodymass	**-0.021**	**-0.038**	**-0.004**

$R^2_m = 0.39$; $R^2_c = 0.80$

Cortisol_both seasons (n = 83)

	estimate	lower 95%-CI	upper 95%-CI
Intercept	3.512	-118.769	125.793
no. of handlings	-3.140	-6.731	0.450
bodymass	**1.703**	**0.228**	**3.178**

$R^2_m = 0.11$; $R^2_c = 0.29$

Cortisone_both seasons (n = 83)

	estimate	lower 95%-CI	upper 95%-CI
Intercept	2.412	2.212	2.613
season_winter	**0.508**	**0.231**	**0.784**
no. of handlings	-0.052	-0.074	-0.030
total duration	**0.0017**	**0.0005**	**0.0029**
season_winter : total duration	**-0.004**	**-0.006**	**-0.002**

$R^2_m = 0.32$; $R^2_c = 0.38$

Cortisol/Cortisone Ratio (n = 83)

	estimate	lower 95%-CI	upper 95%-CI
Intercept	6.003	4.113	7.893
season_winter	-2.264	-4.562	0.034
no. of handlings	**-0.225**	**-0.429**	**-0.020**
gathering duration	-0.063	-0.112	-0.013
handling duration	**0.323**	**0.030**	**0.616**
season_winter : gathering duration	**0.259**	**0.083**	**0.435**

$R^2_m = 0.16$; $R^2_c = 0.30$

Corticosterone_both seasons (n = 83)

	estimate	lower 95%-CI	upper 95%-CI
Intercept	3.006	2.455	3.556
season_winter	0.384	-0.071	0.839
no. of handlings	**-0.074**	**-0.147**	**-0.001**

$R^2_m = 0.06$; $R^2_c = 0.29$

11-deoxycortisol_both seasons (n = 83)

	estimate	lower 95%-CI	upper 95%-CI
Intercept	3.818	2.184	5.452
season_winter	-1.831	-3.957	0.295
no. of handlings	**-0.366**	**-0.554**	**-0.179**
gathering duration	-0.047	-0.091	-0.003
handling duration	0.213	-0.035	0.460
total duration	0.011	0.004	0.018
season_winter : gathering duration	**0.196**	**0.039**	**0.353**
season_winter : total duration	**-0.015**	**-0.024**	**-0.005**

$R^2_m = 0.27$; $R^2_c = 0.49$

17α-hydroxy progesterone_both seasons (n = 83)

	estimate	lower 95%-CI	upper 95%-CI
Intercept	-0.440	-0.945	0.065
season_winter	0.822	0.518	1.127
no. of handlings	-0.048	-0.098	0.002
total duration	**0.003**	**0.0004**	**0.005**

$R^2_m = 0.21$; $R^2_c = 0.57$

Deoxycorticosterone (n = 83)

	estimate	lower 95%-CI	upper 95%-CI
Intercept	-0.460	-1.237	0.317
season_winter	-0.670	-1.619	0.279
no. of handlings	-0.144	-0.228	-0.060
gathering duration	-0.016	-0.036	0.003
handling duration	0.112	0.000	0.225
total duration	0.006	0.003	0.009
season_winter : gathering duration	**0.091**	**0.021**	**0.161**
season_winter : total duration	**-0.007**	**-0.011**	**-0.003**

$R^2_m = 0.20$; $R^2_c = 0.60$

Estimates and confidence intervals (CI) from best model without common intercept

LCC_both seasons (n = 167)

	estimate	lower 95%-CI	upper 95%-CI
seasonsummer	46.168	43.122	49.215
seasonwinter	32.649	29.541	35.756
seasonsummer:no.handlings	**1.313**	**0.887**	**1.740**
seasonwinter:no.handlings	-0.340	-0.920	0.239
seasonsummer:dur.gathering	**-0.129**	**-0.212**	**-0.046**
seasonwinter:dur.gathering	**0.483**	**0.113**	**0.854**

Rectal temperature_both seasons (n = 196)

	estimate	lower 95%-CI	upper 95%-CI
season_summer	40.619	39.242	41.996
season_winter	40.544	39.027	42.061
gathering duration	0.005	-0.001	0.011
total duration	-0.001	-0.001	0.000
bodymass	-0.009	-0.026	0.008
season_summer : no. of handlings	-0.084	**-0.121**	**-0.048**
season_winter : no. of handlings	-0.068	**-0.100**	**-0.037**
season_summer : handling duration	-0.002	-0.054	0.051
season_winter : handling duration	-0.009	-0.022	0.003

Metanephrine_both seasons (n = 83)

	estimate	lower 95%-CI	upper 95%-CI
season_summer	0.437	0.363	0.511
season_winter	0.445	0.371	0.518
no. of handlings	-0.016	-0.024	-0.008
season_summer : gathering duration	**-0.006**	**-0.008**	**-0.004**
season_winter : gathering duration	**0.009**	**0.003**	**0.015**
season_summer : total duration	**0.000**	**0.000**	**0.001**
season_winter : total duration	0.000	0.000	0.000

Normetanephrine_both seasons (n = 83)

	estimate	lower 95%-CI	upper 95%-CI
season_summer	0.633	-0.878	2.144
season_winter	3.636	1.963	5.309
no. of handlings	0.033	0.005	0.061
handling duration	0.036	-0.002	0.074
season_summer : gathering duration	**0.013**	**0.007**	**0.019**
season_winter : gathering duration	-0.024	-0.044	-0.004
season_summer : bodymass	-0.010	-0.029	0.010
season_winter : bodymass	**-0.031**	**-0.051**	**-0.012**

Cortisol_both seasons (n = 83)

	estimate	lower 95%-CI	upper 95%-CI
no. of handlings	-3.109	-6.504	0.287
bodymass	1.745	1.470	2.020

Cortisone_both seasons (n = 83)

	estimate	lower 95%-CI	upper 95%-CI
season_summer	2.412	2.212	2.613
season_winter	2.920	2.695	3.144
no. of handlings	-0.052	-0.074	-0.030
season_summer : total duration	**0.0017**	**0.0005**	**0.0029**
season_winter : total duration	**-0.002**	**-0.003**	**-0.001**

Cortisol/Cortisone Ratio (n = 83)

	estimate	lower 95%-CI	upper 95%-CI
season_summer	6.003	4.113	7.893
season_winter	3.739	2.149	5.329
no. of handlings	-0.225	-0.429	-0.020
handling duration	0.323	0.030	0.616
season_summer : gathering duration	**-0.063**	**-0.112**	**-0.013**
season_winter : gathering duration	**0.196**	**0.039**	**0.353**

Corticosterone_both seasons (n = 83)

	estimate	lower 95%-CI	upper 95%-CI
season_summer	3.006	2.455	3.556
season_winter	3.390	2.840	3.939
no. of handlings	-0.074	-0.147	-0.001

11-deoxycortisol_both seasons (n = 83)

	estimate	lower 95%-CI	upper 95%-CI
season_summer	3.818	2.184	5.452
season_winter	1.987	0.352	3.622
no. of handlings	-0.366	-0.554	-0.179
handling duration	0.213	-0.035	0.460
season_summer : gathering duration	**-0.047**	**-0.091**	**-0.003**
season_winter : gathering duration	**0.149**	**0.013**	**0.285**
season_summer : total duration	**0.011**	**0.004**	**0.018**
season_winter : total duration	-0.003	-0.010	0.003

17α-hydroxy progesterone_both seasons (n = 83)

	estimate	lower 95%-CI	upper 95%-CI
season_summer	-0.440	-0.945	0.065
season_winter	0.382	-0.138	0.902
no. of handlings	-0.048	-0.098	0.002
total duration	0.003	0.0004	0.005

Deoxycorticosterone (n = 83)

	estimate	lower 95%-CI	upper 95%-CI
season_summer	-0.460	-1.237	0.317
season_winter	-1.130	-1.907	-0.353
no. of handlings	-0.144	-0.228	-0.060
handling duration	0.112	0.000	0.225
season_summer : gathering duration	**-0.016**	**-0.036**	**0.003**
season_winter : gathering duration	**0.075**	**0.014**	**0.135**
season_summer : total duration	**0.006**	**0.003**	**0.009**
season_winter : total duration	-0.001	-0.004	0.001
