# Supplementary material for: Getting used to it? Stress of repeated management procedures in semi-domesticated reindeer
Source: BMC Vet Res. 2025 Apr 14;21:268. doi: 10.1186/s12917-025-04718-8 (PMC11995495; doi:10.1186/s12917-025-04718-8)
Supplement: Supplementary file 9 — Supplementary Material 9: Additional table A2: For all stress indices the summary outputs of the summer models are shown, which have been generated to analyse the effects of reproductive status in summer and were based on effects identified for summer in the cross-seasonal models (additional Table A1). Results showing the potential effects of reproductive status were printed black whereas results from independent variables, which were included to correct for their effect in summer identified in the cross-seasonal model, were printed in grey. Significant p-values of seasonal independent variables were highlighted in green and bold; trends of seasonal independent variables were highlighted in green and italics. [file 12917_2025_4718_MOESM9_ESM.pdf]

Calf management in summer

LCC_summer (n = 84)

	Value	Std.Error	df	t-value	p-value
(Intercept)	9.12	0.10	68	90.29	< 0.001
rep.status_calf removed	-0.57	0.11	11	-4.99	**< 0.001**
rep.status_non-reproducing	-0.02	0.15	11	-0.11	0.911
no. of handlings	0.09	0.02	68	5.71	0.000
gathering duration	-0.01	0.00	68	-3.01	0.004

Rectal temperature_summer (n = 126)

	Value	Std.Error	df	t-value	p-value
(Intercept)	39.77	0.16	68	247.13	< 0.001
rep.status_calf removed	0.05	0.24	11	0.22	0.833
rep.status_non-reproducing	0.16	0.31	11	0.52	0.616
no. of handlings	-0.08	0.01	68	-5.88	< 0.001
gathering duration	0.00	0.00	68	1.76	0.083
total duration	0.00	0.00	68	0.26	0.795

Metanephrine_summer (n = 42)

	Value	Std.Error	df	t-value	p-value
(Intercept)	0.33	0.04	25	8.07	< 0.001
rep.status_calf removed	0.07	0.05	11	1.44	0.178
rep.status_non-reproducing	0.10	0.06	11	1.60	0.138
no. of handlings	-0.0108	0.0038	25	-2.84	0.009
gathering duration	-0.0039	0.0009	25	-4.16	0.000
total duration	0.0003	0.0002	25	1.73	0.097

Normetanephrine_summer (n = 42)

	Value	Std.Error	df	t-value	p-value
(Intercept)	-0.29	0.21	25	-1.42	0.168
rep.status_calf removed	0.19	0.19	11	1.01	0.333
rep.status_non-reproducing	0.50	0.25	11	2.00	*0.070*
no. of handlings	0.04	0.02	25	2.18	0.039
gathering duration	0.01	0.00	25	3.14	0.004
handling duration	0.05	0.03	25	1.54	0.136

Cortisol_summer (n = 42)

	Value	Std.Error	df	t-value	p-value
(Intercept)	-48.34	112.16	26	-0.43	0.670
rep.status_calf removed	5.57	19.48	11	0.29	0.780
rep.status_non-reproducing	49.49	25.11	11	1.97	*0.074*
no. of handlings	-2.49	2.56	26	-0.97	0.341
bodymass	2.26	1.44	26	1.57	0.129

Cortisone_summer (n = 42)

	Value	Std.Error	df	t-value	p-value
(Intercept)	3.98	0.32	26	12.48	< 0.001
rep.status_calf removed	0.51	0.31	11	1.62	0.133
rep.status_non-reproducing	-0.21	0.41	11	-0.51	0.619
no. of handlings	-0.13	0.04	26	-2.84	**0.009**
total duration	0.0044	0.0017	26	2.52	**0.018**

Cortisol/Cortisone Ratio_summer (n = 42)

	Value	Std.Error	DF	t-value	p-value
(Intercept)	6.98	1.09	25	6.39	< 0.001
rep.status_calf removed	-0.71	0.74	11	-0.95	0.362
rep.status_non-reproducing	2.40	0.99	11	2.42	**0.034**
no. of handlings	-0.23	0.11	25	-2.15	0.042
gathering duration	-0.08	0.03	25	-2.91	0.007
handling duration	0.33	0.16	25	2.06	0.050

Corticosterone_summer (n = 42)

	Value	Std.Error	df	t-value	p-value
(Intercept)	2.95	0.37	27	7.94	< 0.001
rep.status_calf removed	-0.52	0.54	11	-0.98	0.349
rep.status_non-reproducing	0.84	0.69	11	1.22	0.249
no. of handlings	-0.06	0.05	27	-1.16	0.255

11-deoxycortisol_summer (n = 42)

	Value	Std.Error	df	t-value	p-value
(Intercept)	3.36	1.02	24	3.29	0.003
rep.status_calf removed	-0.31	0.94	11	-0.33	0.746
rep.status_non-reproducing	1.87	1.23	11	1.52	0.157
no. of handlings	-0.37	0.10	24	-3.62	0.001
gathering duration	-0.05	0.02	24	-1.89	0.071
handling duration	0.23	0.15	24	1.54	0.137
total duration	0.01	0.00	24	3.20	0.004

17α-hydroxy progesterone_summer (n = 42)

	Value	Std.Error	df	t-value	p-value
(Intercept)	-0.75	0.38	26	-1.97	0.060
rep.status_calf removed	-0.33	0.48	11	-0.70	0.499
rep.status_non-reproducing	1.17	0.62	11	1.89	*0.086*
no. of handlings	-0.09	0.04	26	-2.05	0.050
total duration	0.005	0.002	26	2.74	0.011

Deoxycorticosterone (n = 42)

	Value	Std.Error	df	t-value	p-value
(Intercept)	-1.20	0.47	25	-2.53	0.018
rep.status_calf removed	-0.16	0.56	11	-0.29	0.778
rep.status_non-reproducing	1.24	0.73	11	1.70	0.116
no. of handlings	-0.13	0.05	25	-2.77	0.010
handling duration	0.13	0.08	25	1.72	0.097
total duration	0.006	0.002	25	3.08	0.005
